# A systematic literature review exploring the application of deep learning in electric vehicles from 2015 to 2025

**DOI:** 10.3389/frai.2026.1826633

**Published:** 2026-08-11

**Authors:** John Vianney Ssennono, Javeed Kittur, Sabah-Ud-Din Waqar

**Affiliations:** 1 Electrical Engineering, Gallogly College of Engineering, The University of Oklahoma, Norman, OK, United States; 2 Engineering Pathways, Gallogly College of Engineering, The University of Oklahoma, Norman, OK, United States; 3 Gallogly College of Engineering, The University of Oklahoma, Norman, OK, United States

**Keywords:** battery management systems (BMS), deep learning, deep learning technologies, deep reinforcement learning (DRL), electric vehicle (EV), electrical engineering, large language models (LLM), long short-term memory (LSTM)

## Abstract

**Introduction:**

Electric vehicles (EVs) are rapidly gaining popularity and global recognition, driven by their reliability, flexibility, simplicity, and scalability. This paper provides a systematic literature review of research at the intersection of electric vehicles and deep learning, aiming to identify current advancements and explore their potential for future scalability.

**Methods:**

A total of 92 publications from 2015 to 2025 were included in the final synthesis phase of the review. These works were categorized into five key themes: data-driven research on electric vehicles and deep learning, societal integration of electric vehicles, implications of electric vehicle adoption, software considerations, and challenges and solutions enabled by deep learning. Crucially, the scope of this synthesis extends into state-of-the-art frameworks spanning 2025 and 2026, evaluating deep learning’s dual footprint in vehicle-level mechanical safety systems, such as machine learning-driven brake-blending policies optimizing regenerative energy capture and fleet-level performance logistics via neural network-driven predictive maintenance optimization.

**Results:**

The findings for each theme and their implications for research and practice are thoroughly discussed. Additionally, a descriptive analysis of research trends shows: (1) a steady increase in publications each year; (2) a majority of contributions originating from China; (3) diverse deep learning approaches being applied to tackle various challenges within the electric vehicle industry; and (4) significant opportunities for the development, testing, and deployment of deep learning technologies and algorithms in the electric vehicle domain.

**Discussion:**

The findings highlight the growing applicwation of deep learning across the electric vehicle domain and demonstrate significant opportunities for the continued development, testing, and deployment of deep learning technologies and algorithms to support future advancements and scalability in electric vehicles.

## Introduction

The transition to the use of renewable energy sources has triggered the creation of a new sector in the automotive and technology sectors. Technology for electric vehicles is a developing subject that offers numerous advantages, such as reduced operating costs (Balan et al., 2022), fulfilling sustainable development goals, and cleaner energy consumption among others. For each technology and its sub-categories, the different resources highlight and discuss their reported advantages and disadvantages when applied to electric vehicles for optimal operation. This involves performance assessment, operational efficiency and stability, resource requirements and scalability, security and privacy features, and applicability and limitations (Ma et al., 2022; Sedhom et al., 2025). These categories have been specifically focused on in this paper to properly analyze the impact of electric vehicles and deep learning on the automotive industry as well as the communities at large. This leads to improved operational efficiency for service providers, cost reduction for stakeholders, alleviating burden on the electric grid, validation of deep learning for complex time series, importance of temporal features and data characteristics, scalability for practical applications, and foundation for future research and development. With these factors, this has led to the number of EVs increasing from 2 million to 5.1 million between 2017 and 2018 (Eddine and Shen, 2022).

Further, the systematic literature review (SLR) is going to look at the different ways research has been prioritized in this sector given the consistent need to improve standards as well as match the speed of technological advancements to match the new efficiency directions. EVs are set to play a vital environmental role due to carbon neutral transportation means, both private and public. With this, EVs can be treated as energy storage devices given their efficiency and flexibility to new technologies, thereby providing great opportunities and services for microgrids (Kaplan et al., 2021). In addition, this has increased the demand for electric vehicles with battery capacity, which has been a challenge to meet by manufacturers due to the limitations of lithium-ion batteries. This has triggered a drop in EV sales and caused friction through transition to EVs despite the upside of embracing the new automotive advancement. State of Charge has become a bigger topic due to how accurate the EVs need to be for estimation of discharging and charging times, making the transition from gas vehicles a seamless move over time (Zhu et al., 2019a, 2019b; Laroui et al., 2019).

In this paper, we aim to explore: What the application of deep learning in electric vehicles is - emerging trends and future directions. The first section of the findings characterizes the sampled articles based on the following attributes: year of publication, publication type and outlet, country of affiliation of the first author, research design utilized, algorithms and technologies used, limitations faced, sampling method and sample size, study population and participant demographics, and future directions pointed to. The second part of our findings provides a synthesis and categorization of research themes across the articles that are looked at.

This study contributes to the existing body of systematic literature reviews by providing a focused and integrative synthesis of research at the intersection of electric vehicles and deep learning over the past decade. Unlike prior reviews that examine these domains independently or focus on specific technical subareas, this review brings together research across multiple countries and application contexts to present a comprehensive view of current developments. By synthesizing trends, applications, and thematic patterns across diverse geographic and technical settings, the study highlights how progress in electric vehicle technologies and deep learning is evolving globally. This cross-context perspective not only captures the current state of the field but also identifies opportunities for collaboration and knowledge exchange across regions and research domains.

## Methods

The PRISMA (Preferred Reporting Items for Systematic Reviews and Meta-Analyses) framework was utilized in this research to guide the SLR process. This framework has three main phases: (1) identification, where the articles are retrieved from different databases using search terms; (2) screening, where the articles are screened by abstract and full text following a set of predefined exclusion criteria; and (3) inclusion, where the final articles included in the review process are analyzed in detail to address a set of research questions revolving around electric vehicles and deep learning (Sundaram et al., 2021; Weiss et al., 2022; Hemdani et al., 2022). We selected the PRISMA framework to guide our SLR process for its transparency and rigorous methodology. The SLR process began by entering the same search term ‘electric vehicles + deep learning’, into seven databases, i.e., Google Scholar, Web of Science, IEEE Explorer, Compendex/ Engineering Village, ERIC, ScienceDirect, and Wiley Online Library. After retrieving a few results that were dedicated to only electric vehicles and deep learning as the main terms, we decided to include articles that involved different specific forms of deep learning like deep Q leaning (Kong et al., 2020; Li et al., 2021) and deep reinforcement learning (Ma et al., 2022; Wang et al., 2022) for a more holistic review of the related data.

The search strategy prioritized conceptual specificity by using the core terms “electric vehicles” and “deep learning” to capture studies at their intersection. To enhance coverage within this focused scope, an iterative screening process was employed, including backward and forward snowballing of selected articles. This approach enabled the identification of additional relevant studies referencing specific deep learning techniques (e.g., neural networks, deep reinforcement learning) that may not have been captured through the initial search string. While this strategy improves coverage, it may still exclude studies framed under broader machine learning terminology, which is acknowledged as a limitation. This review was conducted in alignment with PRISMA guidelines to ensure transparency and rigor in study identification, screening, and reporting, with adaptations made to the search strategy to support a focused and conceptually coherent scope as follows:

With the focus on electric vehicles by different automotive companies and scientific research, there has been a surge in production of electric vehicles due to improvements in their reliability (Kaplan et al., 2021) and fascination in the capabilities of their technologies compared to gas powered cars, for example, immediate responsiveness while driving in addition to features like self-driving (Shafee et al., 2020). We conducted a systematic literature review on how the use of electric vehicles has impacted different communities and how deep learning has been used to improve the user experience. We used Google Notebook LM to extract information from different papers and guide the SLR. Results from this review provide summaries of the main technologies and algorithms used in the deep learning sector (machine learning) to improve how electric vehicles work and impact the lives of the users, for example, battery management, fulfilling Sustainable Development Goals like renewable energy utilization, electric vehicle safety, electric vehicle software capabilities, grid integration, minimizing transportation costs among others.

The studies on electric vehicles involve research related to a broad range of exotic technologies and features including fully autonomous driving, hybrid electric vehicles, increase in driving range, charging station placement among others (Sun et al., 2023; Sotoudeh and Hom Chaudhuri, 2023). This SLR was restricted to applications of different forms of deep learning in the field of electric vehicles and the different challenges and solutions in various locations. Research related to deep learning and electric vehicles independently was excluded as the study aimed to focus on the two areas of the sector rather than the broader aspects of machine learning. This is because Deep learning is a subset of machine learning that uses artificial neural networks with often more than three layers to automatically learn complex patterns from large datasets.

### Data collection

**Figure 1:**
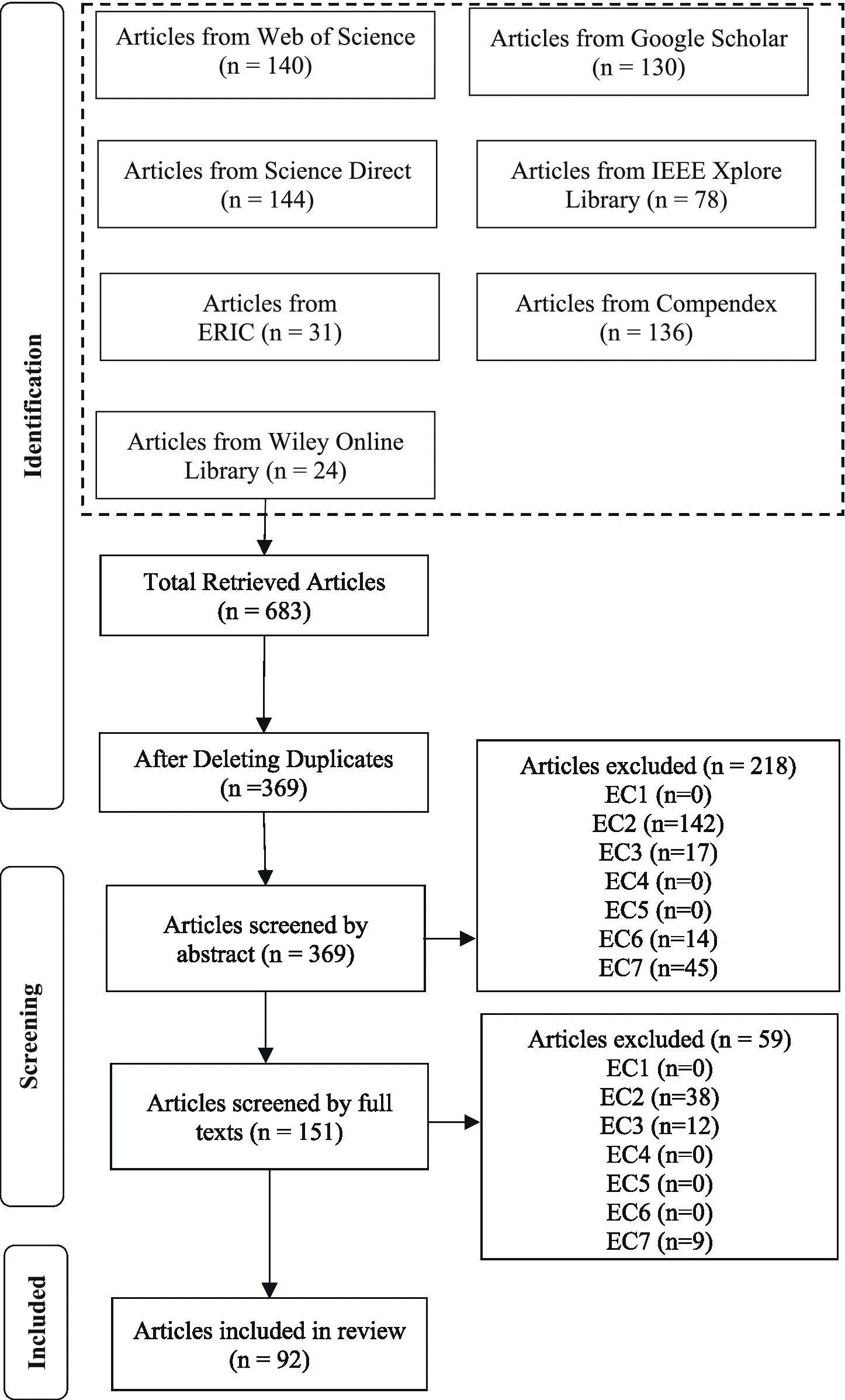
PRISMA flow diagram illustrating the systematic literature review process.

Figure 1 presents the complete article selection process while carrying out the SLR. We eliminated articles that met any of the following seven exclusion criteria (EC) as they did not meet the purpose of the study: EC1: Published before 2015; EC2: Not necessarily has a balanced focus on factors directly affecting electric vehicles and deep learning. The article is too focused on specific fields other than electric vehicles and deep learning in general; EC3: Articles that are short papers or working progress; EC4: Articles that are published in other languages other than English; EC5: For the final sources, assess the quality of the papers, low quality is excluded; EC6: Identical articles from the same search engine; EC7: Inaccessible full record of the selected articles, i.e., the articles that are not wholly accessible for review.

**Figure 2:**
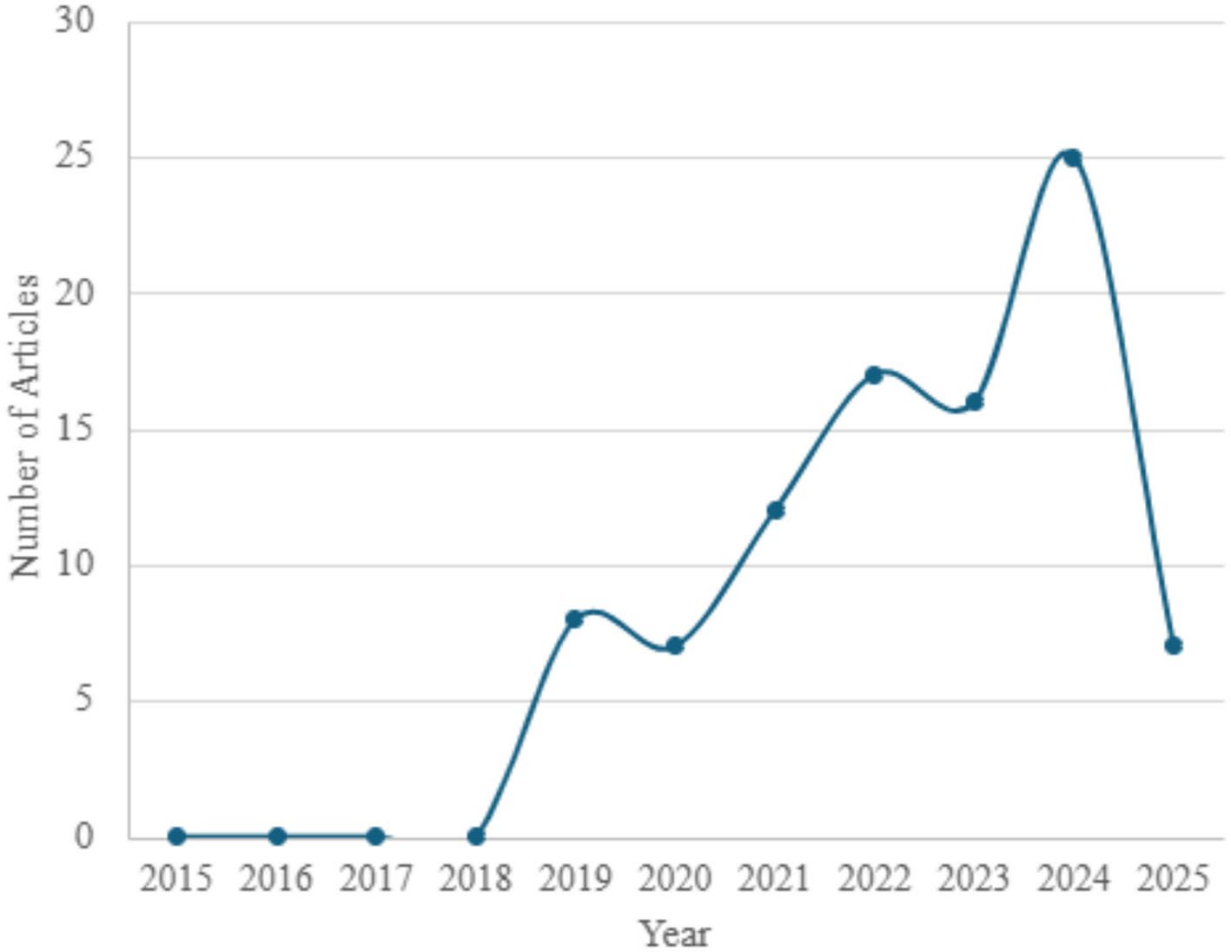
Number of sampled articles between 2015 and 2025.

As indicated in Figure 2, 369 articles were retrieved from the seven databases using one search term, ‘electric vehicles AND deep learning.’ To conclude the identification phase, the first author removed duplications among articles pulled from various databases and then completed the initial screening of abstracts. After, the full remaining articles were reviewed to eliminate those that were easily identifiable by meeting one or more of the exclusion criteria listed. The articles were screened individually by the first author based on whether they explored the relationship between deep learning and electric vehicles as follows:

- Stage 1: Broad Title Screening: The initial keyword search targeted core terminologies across multiple databases. The resulting literature pool was first filtered based strictly on article titles. Titles were required to explicitly contain “electric vehicles + deep learning,” or terms representing topics directly related to these two primary terminologies.
- Stage 2: Abstract-Level Evaluation: For all articles that passed the initial title-based filter, their abstracts were comprehensively reviewed to evaluate conceptual alignment with the core focus of the study.
- Stage 3: Comprehensive Full-Text Review & Strict Exclusion: The remaining articles were retrieved for full-text evaluation. At this stage, strict content-driven exclusion criteria were enforced to safeguard the quality of the data. Specifically, any papers identified as ongoing research, or articles that only provided extended abstracts without complete, peer-reviewed methodologies and full-text findings, were dropped from the final list, even if their titles originally matched our initial criteria.
- Final Synthesis Count: Following this exhaustive filtration process, 92 papers met all baseline criteria and were chosen for the literature synthesis. In addition to these 92, the state-of-the-art papers recommended during this interactive peer-review process have been integrated, further elevating the fullness of our research.
- Author Validation and Quality Assurance: To eliminate individual bias and verify the rigor of the selection process, the second and third authors independently audited the chosen articles. Following cross-checking and collective verification, all authors reached a unanimous consensus that the final article count is highly substantial for a comprehensive review of this domain and contains the precise technical depth needed to answer the research questions posed.

### Data analysis

With the 92 articles selected, the authors followed a six-step process to synthesize and create meaning from the publications. To ensure consistency in the study selection process, the first and third authors independently reviewed an initial subset of studies (*n* = 10). They then compared their evaluations and resolved any discrepancies through discussion. After achieving alignment, the remaining studies were divided equally and reviewed independently. Inter-rater reliability was assessed using Cohen’s Kappa, which yielded a value of 0.86, indicating strong agreement between the reviewers.

To support the efficiency of the review process, AI-based tools (e.g., Google NotebookLM) were used for preliminary summarization of selected articles. These summaries served only as an initial reference and were not used directly for analysis. Each AI-generated summary was carefully reviewed and verified against the original full-text article by the authors to ensure accuracy and completeness. Any inconsistencies or misinterpretations were corrected through manual review and discussion. To further ensure rigor, the summarized information for each article was cross-checked by an additional author to confirm consistency and eliminate duplication. All coding, categorization, and thematic synthesis were conducted manually by the authors, independent of AI tools. The role of ChatGPT was limited to improving language clarity and readability, and it did not contribute to data interpretation or analytical decisions.

These steps ensured that the analysis remained transparent, reproducible, and grounded in author-driven interpretation while minimizing potential biases associated with AI-assisted summarization. Additionally, inter-rater reliability (Cohen’s Kappa = 0.86) indicates strong agreement between reviewers, further supporting the validity of the analysis.

The following information was recorded for each article by each author: year of publication; publication type (for example, conference proceeding or journal publication) and publication release; research question being answered by the paper; country affiliation of the first author; methodology of each paper; stakeholder involved in each study; research focus and research methods applied; study populations and participant demographics; sampling methods and range of sample sizes; research findings; algorithms/ technologies used; implications of each study; limitations; future work pointed out; and methods of investigation and evaluation.

Further, the first author generated codes with comparison between all the selected 94 articles to come up with five themes encompassing all the common patterns in place. At first, 24 codes were generated: EV data handling, EV data utilization, EV energy management, grid integration, human driving behavior, EV safety measures, fault diagnosis, operational efficiency, forecasting accuracy, cybersecurity, fulfilling SDGs (Sustainable Development Goals), EV software optimal capabilities, environmental planning, EV software capabilities, design evaluation, renewable energy utilization, model optimization, range prediction accuracy, minimizing EV costs, EV infrastructure improvements, battery hardware management, EV software development, EV safety controls, and EV policy analysis.

With further review of the mentioned codes, they were all uniquely clustered together to come up with the matches to analyze the best descriptions of the themes by the first and second author: electric vehicles and deep learning research data results, implications of electric vehicle and deep learning research, electric vehicle societal integration, implications of electric vehicle societal integration, software implications, and challenges and solutions with electric vehicles using deep learning. This was done by reading through each article summary of “research question being answered’ and their respective findings to categorize which codes mainly resonated with each article.

The thematic categories were developed using an inductive, iterative approach aligned with reflexive thematic analysis. Codes were generated from the data and progressively grouped into broader patterns, which were then reviewed and refined to develop coherent themes. Themes were defined to capture distinct analytical dimensions of the literature, with attention to internal consistency and conceptual clarity. Given the interdisciplinary nature of the topic, themes were not treated as mutually exclusive; instead, they represent interconnected domains, and individual studies may contribute to multiple themes. This approach reflects the complexity of the research landscape while maintaining analytical coherence.

Data analysis in this paper is presented in two parts. The first phase uses descriptive statistics to examine the trends in the 92 articles that remained for final investigation. The second part presents the qualitative analysis of these articles across the five identified themes (Electric Vehicles and Deep Learning Research Data Results, Implications of Electric Vehicle and Deep Learning Research, Electric Vehicle Societal Integration, Implications of Electric Vehicle Societal Integration, Software Implications, and Challenges and Solutions with Electric Vehicles using Deep Learning).

## Strengths and limitations

This SLR provides a holistic picture of research on electric vehicles and deep learning by assessing the trends and current state of knowledge in this sector. Each theme generated as part of the study is complemented by implications for both practice and research meant to provide practitioners and researchers in the field of electric vehicles with specific and applicable guidance on how to use deep learning as a tool for improving their work. The findings of this study greatly expand the current understanding of research in the field of deep learning and electric vehicles as they assist in grouping the strengths of existing research and pointing out opportunities for future research.

In the line of limitations, we selected the final set of 92 articles based on exclusion criteria that did not include a metric of quality or uniqueness of information therein. In alignment with other SLRs within electric vehicles and deep learning (e.g., Zhang et al., 2022; Eddine and Shen, 2022; Sun et al., 2023), we used seven databases that we expected to contain many journal and conference articles focused on electric vehicles and deep learning research; we hope this limitation was eliminated through using these separate and highly reputable databases. We also excluded working progress papers, short papers, and other technical reports in our search, which may have limited the scope of information covered within the SLR. The search terms we used in this study focused on the intersection of electric vehicles, deep learning, and specific areas of interest like renewable energy utilization, grid integration, operational efficiency, which may have excluded other articles and themes relevant to engineering education that might have emerged using different combinations of these and other terminologies. Since we did not include articles published before 2015 and after July 2025, the review might miss other impacts of deep learning and new milestones in the electric vehicle sector that practitioners and researchers might find appealing and relevant but were not yet published. Because we limited the articles to English, they may have only denoted a portion of the global publications on electric vehicles and deep learning.

This review did not employ a formal risk-of-bias or quality scoring framework. The primary objective of the study was to provide a broad synthesis of research trends and thematic patterns rather than to evaluate the effectiveness or methodological rigor of individual studies. Given the heterogeneity of study designs included in the review (e.g., simulation-based, experimental, and descriptive studies), applying a standardized quality assessment tool was not considered appropriate. Instead, structured inclusion and exclusion criteria were used to ensure relevance and baseline quality. However, this approach may result in all studies being treated equally regardless of methodological rigor, which is acknowledged as a limitation.

### Findings

A detailed summary of various deep learning-related technologies used in solving challenges in the electric vehicle industry and implications of these actions to the communities involved at large over the decade 2015–2025 are first presented. In addition, we provide descriptions, exemplary studies, and research and practice implications for the five themes identified from synthesizing the final 92 articles.

## Descriptive findings related to publication trends

### Publications per year

Between 2015 and 2025, there was a general increase in the number of articles published on electric vehicles and deep learning research per year, increasing gradually between 2015 and 2018, booming in 2019 then reaching a high in 2022, and dropping off in 2023 and between 2024 and 2025. There is a sudden drop in publications from 2024 to 2025; this is due to the time frame used to carry out research. The research was done over just the first 5 months in 2025, which means few articles were looked at compared to a whole scale of a year for other years covered.

### Publication type and publication outlet

**Figure 3:**
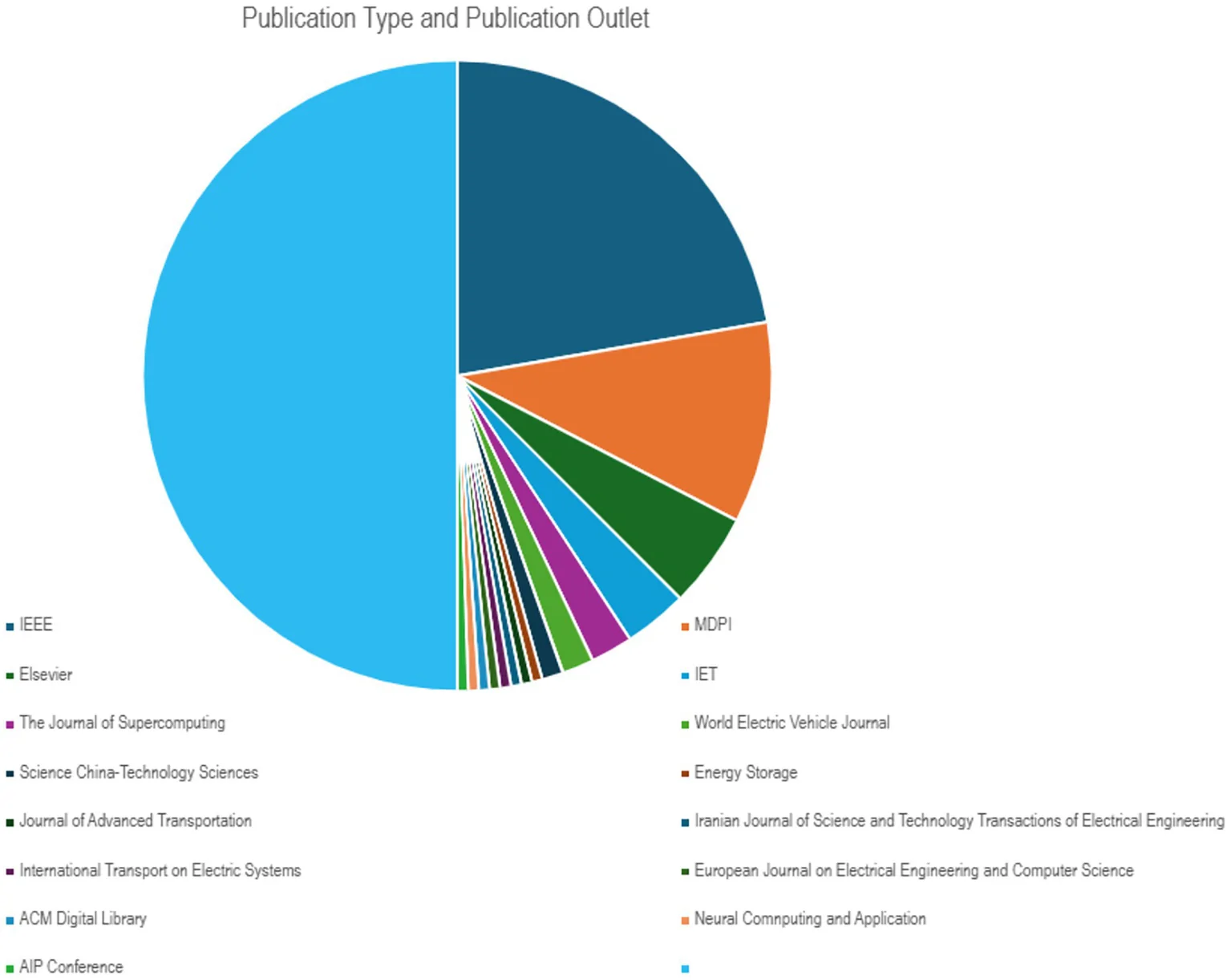
Publication type and publication outlet of sampled articles.

Among the 92 articles reviewed in this study, 53.3% were published as journal articles focused on deep learning, 46.7% were conference articles, and 1.1% were systematic reviews of existent work in the field. A visual representation is presented in Figure 3. In this study, the distribution of articles is balanced. Most of the sampled articles used in this systematic review were published by the Institute of Electrical and Electronics Engineers (IEEE) (44.6%), then 20.7% by the MDPI, 9.8% by the Elsevier, 6.5% by IET, 4.3% by The Journal of Supercomputing, 3.3% by World Electric Vehicle Journal, and the remaining are from 9 other publishers like European Journal on Electrical Engineering and Computer Science, Iranian Journal of Science, ACM Digital Library, AIP Conference, and Journal of Advanced Transport each having 1 article.

### Country affiliation of first author

**Figure 4:**
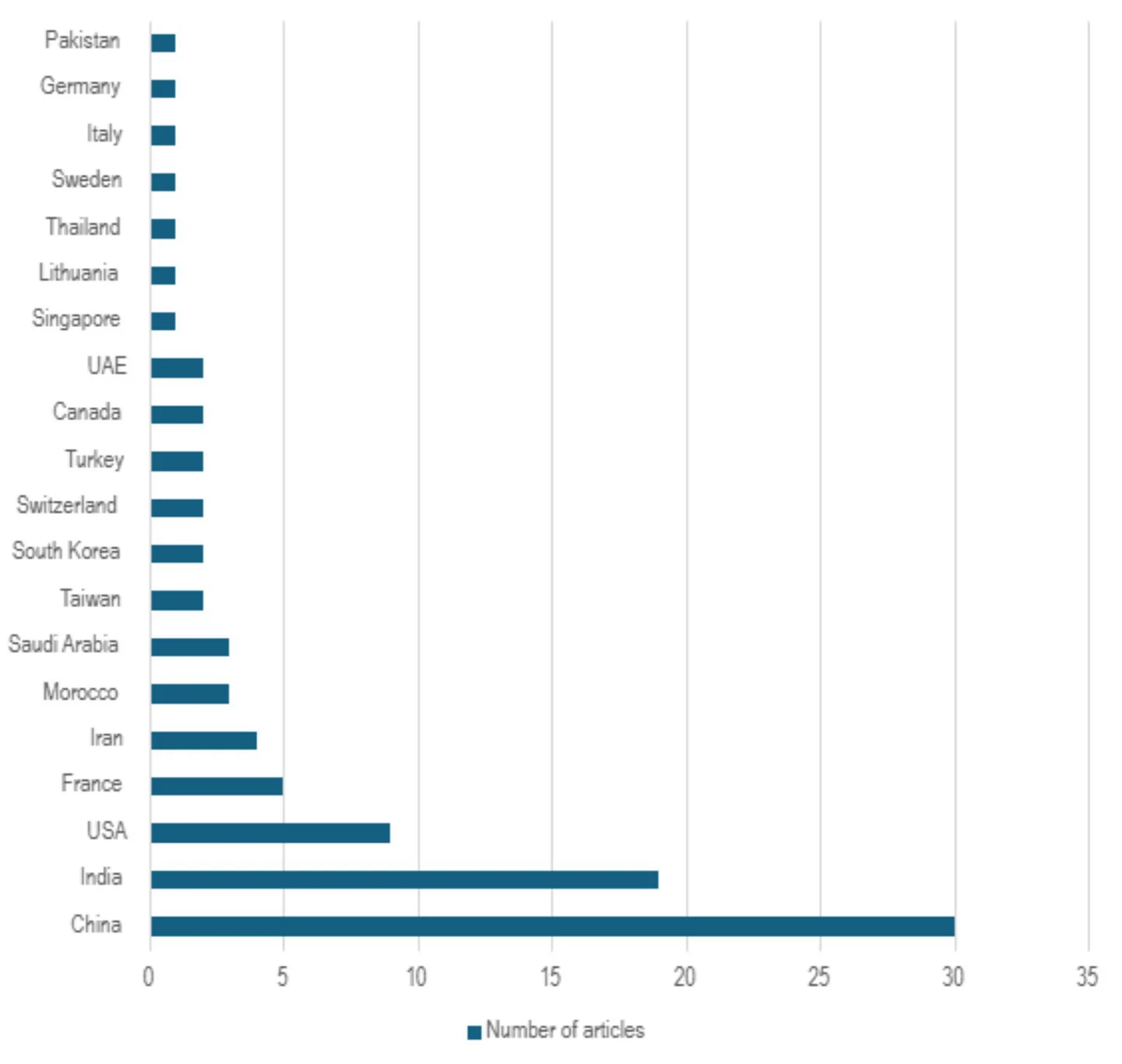
Number of sampled articles by the country of the first author’s affiliation.

Figure 4 shows that the articles in this systematic review featured first authors from 25 different countries. Most of the first authors affiliated with China (35.2%), then, India (17.2%), USA (9%), Iran (4.9%), after, Morocco, France, and Saudi Arabia had 3.3%. Taiwan and South Korea had 2.5%, and Malaysia, Germany, Switzerland, Pakistan, Turkey, Egypt, and Canada had 1.6%. The remaining 10 countries each had 0.8% of the 92 articles the literature review focuses on. We acknowledge that the high occurrence of electric vehicle and deep learning research is published by authors with affiliations in China which is due to the exponential increase in production and development of electric vehicle technology in China. This has given more people in the country more exposure to this technology, giving an explanation for the large numbers in publications compared to any other country. This is to serve as a basis for future research and bring together more collaboration around the world in this sector given the common language. Furthermore, practitioners in other countries may engage and invest in electric vehicles and deep learning but have different pressures, incentives, or research support infrastructure to publish on this work.

### Research foci and research methods

Table 1 summarizes the different research focus and methods used in the 92 sampled articles. Four main types of articles were found in the research: articles focused on societal integration (65.2%), articles focused on fundamental research, i.e., general research on the electric vehicles and deep learning collaboration (20.7%), articles focused on software/new technology development and implementation (8.7%), and literature reviews (5.4%). The studies marked as descriptive in the table below did not include original data collection and analysis.

**Table 1:** Research foci and methods.

#	Research foci	*N*	%	Research methods	*N*	%
1	Societal integration	60	64.52	Qualitative Quantitative Qualitative and quantitative Descriptive	8 33 15 5	8.7 35.5 16.3 5.4
2	Fundamental research	19	20.43	Qualitative Quantitative Qualitative and quantitative Descriptive	4 6 8 1	4.3 6.5 8.7 1.1
3	Software/ new technology implication	9	9.68	Qualitative Quantitative Qualitative and quantitative Descriptive	1 – 6 2	1.1 – 6.5 2.2
4	Systematic literature review	5	5.38	Descriptive	5	5.4

The three classified systematic literature reviews in electric vehicles and deep learning cover topics such as providing a comprehensive evaluation of new advancements in battery thermal management systems for electric vehicles (Jahromi et al., 2024), common deep learning techniques used for state of health estimation of lithium-ion batteries in electric vehicles, their application, challenges, solutions and future directions (Liu et al., 2025; Trivedi et al., 2022), and centralized and decentralized control approaches for electric vehicle battery management (Shokati et al., 2024).

Further, for the 60 articles focusing on the societal integration of electric vehicles being a new advancement in many communities, they mainly used qualitative and quantitative research methods. The qualitative research under this category collected data from the populations investigating how convenient they find the use of electric vehicles (Duan et al., 2023), the impact of having electric vehicles as their main transportation means and investigating ways to make electric vehicles more efficient and reliable to the public at large (Liang et al., 2024). Likewise, the quantitative studies surveyed mainly service providers and data stations like charging stations, programmers, and electric vehicle companies investigating how well equipped the electric vehicles are to satisfy the demands of the population. Then, research studies focused on both quantitative and qualitative research merged the impacts of electric vehicles on the users with the uprising technology advancements to find a correlation between the two (Huang et al., 2024).

Nine articles concentrated on software/new technology development and implementation, but 12 articles of the 15 covered both quantitative and qualitative research. In these articles, one study focused on developing an adaptive integrated thermal and energy management system for hybrid electric vehicles using multi-agent deep reinforcement learning to optimize fuel consumption, reduce battery degradation, and maintain cabin comfort while adapting to varying driving profiles and thermal loads (Khalatbarisoltani et al., 2024). Studies using both quantitative and qualitative methods collaboratively used data collected to solve qualitative challenges facing usage of electric vehicles. In addition, two articles were descriptive, one covering developing a DRL-based torque distribution strategy to improve energy efficiency and active safety in DDEVs. The other article focuses on designing a learning-based energy management strategy for hybrid battery systems that optimizes energy distribution, minimizes energy loss, and enhances electrical and thermal safety in battery electric vehicles (Li et al., 2021).

Finally, 20 articles were fundamental research papers, published to widen the general knowledge in the sector of electric vehicles and increase the usage of deep learning as a tool in this field. All papers used quantitative, qualitative, as well as descriptive methods to expound the knowledge they are adding to this field. Specifically, most papers employed a combination of qualitative and quantitative methods where they collected data and performed analysis using more advanced statistical techniques. Also, papers under this category were more likely to use public data like how busy EV charging stations are to track progress in the grid integration sector.

### Study populations and participants’ demographics

Table 2 shows the study populations included among the 92 articles in this systematic review. Thirty-five articles used population participation in their respective areas to carry out research for data collection, fifty-seven articles did not involve respective communities directly, and only five articles were systematic reviews, i.e., did not need primary data collection. A total 52 of the 92 articles were neither population-related nor systematic reviews. In the SLR, we found out that the 35 (38.0%) articles that directly involved communities were more accurate at solving the challenges faced by the populations compared to the rest of the 57 (62.0%) articles based on research findings.

**Table 2:** Population demographics.

#	Communal engagement	Number of articles	Percentage (%)
1	No population involved	52	56.99
2	Population involvement	35	37.63
3	Systematic review	5	5.38

### Sampling approaches and sample sizes

Table 3 highlights the sampling methods and sample sizes for the studies referenced above, categorized by research foci (refer to Table 1). Sample size ranges in Table 3 are based on studies for which sample sizes were emphasized. The results reveal a tendency for studies focusing on software/ new technology implications to draw data from less participants prompting them to rely on smaller sample sizes due to the need for extensive individual research needed to be done over long time periods before large sample application. On the other hand, research around societal integration, deep learning and data research, and challenges and solutions to using deep learning in electric vehicles rely on larger sample sizes from which more data points are available to conduct associated analyses (e.g., consumer satisfaction, energy management, grid integration). These results point out an opportunity to increase the generalizability of the collected results from the sample communities used by engaging multiple drivers for more accurate results as some data is subjective but also averaging calculated results, like for application of deep learning technologies, over a large sample space to maximize accuracy. Further, this increases the chance of collaboration from like-minded stakeholders to achieve similar goals despite inputting individual efforts into the field.

**Table 3:** Sampling approaches and sample sizes.

Research foci	*N*	%	Sampling method	*N*	%	Sample size: range (median)
Software/ new technology implications	11	12.9	Multiple Single	16 0	100	0–10,000
Societal integration	60	64.52	Multiple Single	76 0	100	0–2,000,000
Does not apply (Not research; descriptive)	21	22.58	None	0	0	0

### Comparative analysis of deep learning models in electric vehicle applications

A cross-study synthesis of the reviewed literature reveals clear methodological trends in the application of deep learning within electric vehicle research (see Table 4). Approximately 60–70% of the analyzed studies employ time-series-based models, particularly LSTM and GRU architectures, reflecting the inherently sequential nature of electric vehicle data (e.g., battery performance, charging cycles, and load demand).

**Table 4:** Comparative overview of deep learning approaches in electric vehicle research.

Model type	Typical applications	Key strengths	Key limitations	Common evaluation metrics
CNN (Convolutional Neural Networks)	Fault detection, visual diagnostics, battery defect analysis	Strong spatial feature extraction; effective for image-based data	Limited capability for temporal dependencies; requires large, labeled datasets	Accuracy, Precision, Recall
RNN (Recurrent Neural Networks)	Sequential modeling, energy consumption prediction	Captures temporal patterns in sequential data	Prone to vanishing gradients; lower performance for long sequences	Accuracy, MSE
LSTM (Long Short-Term Memory)	State-of-charge estimation, battery health prediction, load forecasting	Captures long-term dependencies; high predictive performance in time-series tasks	Computationally intensive; complex hyperparameter tuning	MSE, MAE, R^2^
GRU (Gated Recurrent Unit)	Real-time prediction, load forecasting, fault detection	Faster training than LSTM; comparable performance in many tasks	Slightly reduced modeling flexibility compared to LSTM	MSE, MAE
DRL (Deep Reinforcement Learning)	Energy management, charging optimization, scheduling	Adaptive decision-making in dynamic environments; supports optimization tasks	High training complexity; convergence instability; requires large datasets	Reward value, convergence rate

Among these approaches, LSTM models are most frequently used in applications such as state-of-charge estimation and battery health prediction, where capturing long-term temporal dependencies is critical. Across multiple studies, LSTM-based models consistently demonstrate improved predictive performance, often reflected in lower error values (e.g., mean squared error) compared to traditional machine learning methods. GRU models show comparable predictive capability while offering reduced computational complexity, making them more suitable for real-time or resource-constrained applications.

CNN-based approaches are less frequently used and are primarily applied to spatial or image-based tasks such as fault detection and diagnostics. In contrast, deep reinforcement learning (DRL) approaches are emerging in optimization-focused applications, including energy management and charging strategies, where adaptive decision-making under dynamic conditions is required.

Despite these advancements, the analysis identifies several critical limitations across literature. First, there is substantial variability in evaluation metrics, with studies reporting performance using different measures (e.g., accuracy, MSE, MAE, R^2^), which limits direct comparability. Second, a significant portion of studies rely on simulation-based or synthetic datasets, with limited validation using real-world data. Third, there is a lack of standardized benchmarking frameworks, making it difficult to systematically evaluate the relative performance of different models.

These findings highlight a key gap in the field: the need for standardized datasets, consistent evaluation practices, and benchmarking protocols to improve reproducibility and enable more meaningful cross-study comparisons. Beyond technical implications, these findings have important relevance for engineering education and research training. The dominance of specific model types (e.g., LSTM and GRU) and the lack of standardized evaluation practices suggest a need to better prepare students and researchers to critically assess model selection, interpret performance metrics, and understand limitations related to data quality and reproducibility. Integrating these insights into engineering curricula and research training can support the development of more rigorous, transparent, and transferable approaches to applying deep learning in complex, real-world domains such as electric vehicles.

## Thematic analysis: descriptions, examples and implications

In this section, we explore the five determined themes (Electric Vehicles and Deep learning and Data Research, Electric Vehicle Societal Integration, Implication of Electric Vehicle Societal Integration, Software Implications, and Challenges and Solutions with Electric Vehicles using Deep learning) in depth. We introduce the theme, explain how articles grouped under the theme they are related to, present two exemplar studies identified based on the relatively (among the category within the theme) strong connection to the theme, and conclude within a summary of implications of the theme for future research and practice. Exemplars explicitly focused on the topic of the theme were chosen in comparison to other papers that may have only touched upon the topic. The presentation of the thematic analysis has been inspired by several recently published SLRs (Nguyen and Kittur, 2024; Kittur et al., 2024; Nguyen and Kittur, 2025). In Table 5, each of the five themes is defined, the codes mapped to that theme (see Appendix A for code definitions), and the count of the papers that were categorized in relation to the specific theme are presented. Appendix B provides a table mapping of articles to themes; notably, an article could be mapped to more than one theme.

**Table 5:** Mapping of final articles based on identified themes.

Themes	Definition	Code	#
Electric vehicles and deep learning data research	Topics related to Electric vehicles and deep learning research data. This includes simulations and raw data collection which are evaluated based on each resource theme.	EV data handling EV data utilization Model optimization Design evaluation	28
Electric vehicle societal integration	Topics related to the implementation of electric vehicles in the chosen communities where the different research papers are focused. This looks at the goals of using electric vehicles and how their real-world implementation has affected the communities at large, i.e., energy costs, and electric vehicle safety among others.	EV safety measures EV energy management Human driving behavior Minimizing EV costs Operational efficiency Forecasting accuracy Grid integration EV infrastructure improvements Battery hardware management	86
Software implications	Topics related to the software side of electric vehicles play a large role in making EVs. This could point strongly in the electric vehicle’s functionality and safety for the users. Further, this could mean improvements in the electric vehicle industry as well as drawbacks.	Cybersecurity EV data handling EV data utilization Model optimization State of charge estimation EV software development EV safety controls	39
Implications of electric vehicle societal integration	Topics related to the results of electric vehicles being used in the community at large. This theme looks at the evaluation and results of using electric vehicles in general based on the reported outcomes, i.e., pros and cons of using EVs.	Fulfilling SDGs (Sustainable Development Goals) Renewable energy utilization Minimizing EV costs EV safety controls EV policy analysis Range prediction accuracy	59
Challenges and solutions with electric vehicles using deep learning	Topics related to the challenges and solutions that have been brought by using electric vehicles and what solutions have been proposed by deep learning (software). This could include efficiency, safety, and fault detection, which could enhance the overall functionality of electric vehicles at large.	Fault diagnosis Grid integration Battery hardware management Operational efficiency Forecasting accuracy EV energy management EV safety measures Cybersecurity	42

**Figure 5:**
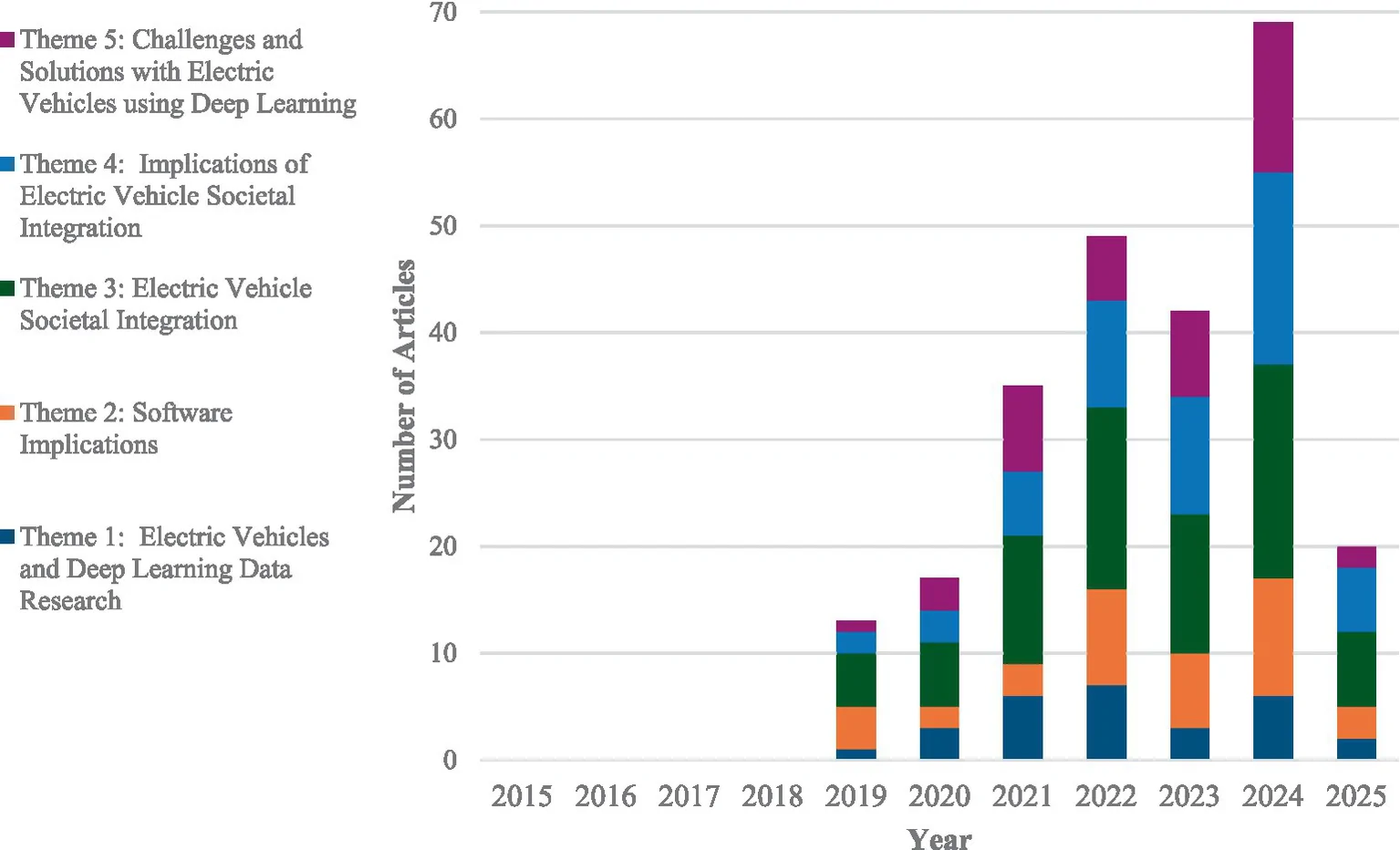
Article publication by theme from 2015 to 2025.

Figure 5 shows the rate at which the different themes have been visited in different research studies done between 2015 and 2025. Starting from the year 2018, research papers became more rampant due to the popularity of electric vehicles increasing in the car automotive market. The theme of Electric Vehicle Societal Integration has seen steady revisiting different researchers over time. In addition, the theme of Implications of Electric Vehicle Societal Integration and the theme of Challenges and Solutions with Electric Vehicles using Deep Learning have seen a consistent trend in various articles over the years, respectively.

### Theme 1: electric vehicles and deep learning data research

Electric vehicles and deep learning research involve the different research projects that have been conducted in line with improving electric vehicle transportation. This research has been applied in different fields including real world usability, electric vehicle technology, and electric vehicle cost minimization among others. Eighteen articles (e.g., Ha et al., 2021; Lin et al., 2022; Hu T. et al., 2021; Hu S. et al., 2021; Alhazmi, 2024; Zhu et al., 2019a, 2019b; Erüst and Taşcıkaraoğlu, 2024; Malek et al., 2021; Mukherjee and Chakraborty, 2022; Khalatbarisoltani and Han, 2024; Aduama et al., 2023; He et al., 2022) have focused on the data handling, model optimization and design evaluation of electric vehicles and providing a firm ground to build on in future work. Under this theme, the focus is fault detection using data results, testing different findings with real world applications and simulations, and management and denoting different research data results for future work.

Then, in the other two articles (e.g., Hasib et al., 2024; Kalaimathi et al., 2025), there is a focus on how electric vehicle data that is collected is utilized during the research. Hasib et al. (2024) emphasized the sensitivity to data quality and quantity, “black-box” nature of deep neural networks, performance in extreme environmental conditions, reliance on simulation-based work and data availability, computational demands and deployment challenges for more complex models, and need for continuous refinement and validation. This tested the electric vehicle sample design and model optimization for real world usage, which is a cornerstone for improvement of the sector at large.

#### Exemplar studies

The following exemplar studies were chosen because they focused on the research that was strongly data collection driven to achieve breakthrough results to lay a foundation in the electric vehicle sector. In a first study, Li et al. (2018) aims at how to accurately determine the vehicle states in autonomous four-wheel independently actuated (FWIA) electric vehicles despite the inherent challenges of sensory signals being corrupted. The paper looks at the brain-inspired proprioceptive system concept, learning the forward model using deep learning (deep recurrent neural networks), network architecture, training process and data collection, simulated impairments, data fusion using unscented Kalman predictor, simulation and performance evaluation, evaluation metrics, and application in control. The selected paper primarily focuses on developing a brain-inspired proprioceptive system to accurately determine vehicle states in autonomous FWIA electric vehicles. This system is designed to overcome the significant challenges posed by sensory signals being corrupted by time-varying delays and measurement of noises in real-world implementations. The aim is to design a proprioceptive system for autonomous FWIA electric vehicles that can effectively compensate for time-varying delays in the feedback loop and simultaneously suppress measurement noises, drawing inspiration from the human sensorimotor loop. This system aims to improve vehicle state estimation and, consequently, the performance of motion control systems like yaw rate tracking.

In the same manner, the research question being answered by Trivedi et al. (2022) implicitly addresses the limitations of existing electric vehicle fault detection systems, particularly concerning the comprehensive identification of diverse faults, and the security and integrity of fault data. To achieve these aims, the paper proposes a deep learning and blockchain-based EV fault detection framework. It leverages convolutional neural networks for air tire pressure fault detection and long short-term memory models for temperature anomaly detection and battery state of health prediction. The investigation uses publicly available datasets for each fault type: air tire pressure fault - an image dataset of full and flat tires and thermal/temperature fault - the numenta anomaly benchmark dataset for outlier detection.

#### Research implications

A primary area for future study is developing electric vehicles and deep learning data research. The current study focused on compensating for large, time-varying network delays in the feedback loop (from sensors to the Vehicle Control Unit - VCU) (Li et al., 2018). Further, the system draws inspiration from the human brain’s sensorimotor loop, which combines sensory feedback with internal predictions to estimate body and environmental states. When integrated with a control algorithm, such as Second-Order Sliding Mode Control, the proprioceptive system substantially improves vehicle motion control performance. The study acknowledges several limitations and points out crucial areas for future research: The current system relies on an offline training method for the RNN, which needs to evolve into online learning to adapt to real-time changes in vehicle parameters like mass and road friction coefficients, as vehicle dynamic models are time-varying.

#### Practical implications

A few studies suggest different pathways that could be applied to improve the electric vehicle sector using various data collected from research studies. Li et al. (2018) paper highlights the successful development and validation of a brain-inspired proprioceptive system designed to address the critical challenges of feedback delays and measurement noises in autonomous four-wheel independently actuated electric vehicles. Another study points out that its proposed framework offers a robust and comprehensive solution that simultaneously delivers improved accuracy, high security, reduced data storage costs, high reliability, and enhanced efficiency for electric vehicle fault detection. This significant contribution supports the ongoing transition towards an intelligent transportation system by ensuring safer and more reliable EV operation (Trivedi et al., 2022). Further, another chosen paper proposes the application of a recent metaheuristic algorithm, the Evolutionary Mating Algorithm, to optimize the parameters of a Deep Learning model (specifically a Feed-Forward Neural Network - FFNN) for State of Charge estimation, creating the EMA-DL model (Sulaiman and Mir, 2024). Together, these studies provide research results from their respective specifications to modify how data in electric vehicles are collected and utilized for better understanding. This will improve how electric vehicles are built to meet the needs of different consumers based on their specific needs.

### Theme 2: software implications

In the process of carrying out electric vehicle research, there is a focus on how deep learning research in electric vehicles is implied. Under this theme, 57 articles (e.g., Peyravi et al., 2025; Mei et al., 2023; Khalatbarisoltani et al., 2024; Huang et al., 2024; Douaidi et al., 2021; Liang et al., 2024; Zhao et al., 2024; Hasib et al., 2024) look at the model optimization and software development of electric vehicles based on the available research that has been carried out in various parts of the world.

Five articles (e.g., George and Sivraj, 2021; Sulaiman and Mir, 2024; Sedhom et al., 2025; Jahromi et al., 2024; Latif et al., 2023) look at model optimization, specifically, renewable energy utilization and range prediction accuracy. Cybersecurity is talked about in 22 articles (e.g., Shafee et al., 2020; Bergies et al., 2024; Salunkhe et al., 2022; Raj and Sakthivel, 2024; Lee and Choi, 2024) where they point out fault diagnosis methods using different software tools and data protection for electric vehicles for safety reasons.

#### Exemplar studies

George and Sivraj (2021) study aimed at how a deep learning-based, optimized system using sequential neural networks can be developed to accurately predict the driving range of electric vehicles, considering the impact of various factors such as driver behavior, exploitation environment, battery parameters, and auxiliary loads, to address the challenge of ‘range anxiety’ experienced by electric vehicle consumers. The analysis reveals that driver behavior, exploitation environment, battery parameters, and auxiliary loads significantly impact the driving range of electric vehicles. A comparative study of five different sequential models (Dense layers, Simple Recurrent Neural Networks, Long Short-Term Memory, Bidirectional Long Short-Term Memory (Bi-LSTM), and Gated Recurrent Units) was conducted, and the Bi-LSTM model demonstrated superior performance among the tested sequential models. When the trained Bi-LSTM model was validated on a new, unseen drive cycle (FTP-75), it initially yielded an R-squared score of 0.9961, a Mean Square Error of 9.9603 km, and a Mean Absolute Error of 2.8189 km. The paper concludes by highlighting the successful development and evaluation of a regression-based neural network model designed to predict the driving range of electric vehicles.

Shafee et al. (2020) discuss the impact of electric vehicles reporting false State of Charge (SoC) values on existing charging coordination mechanisms, how does this behavior affect both lying and honest electric vehicles, and can a deep learning model be effectively devised and deployed to accurately detect lying electric vehicles that send false SoC data to a charging coordinator, thereby ensuring fair charging coordination. The evaluations confirm that reporting false (lower) SoC values is beneficial for lying electric vehicles because it significantly increases their chance of charging before their charging requests expire. In contrast, honest electric vehicles are harmed by the presence of lying electric vehicles. They may not be able to charge at all, or they may experience significant delays, being required to charge late. The selection probability for lying electric vehicles remains consistently high until the number of lying electric vehicles reaches a certain value, after which it starts to decrease, as even some lying electric vehicles may not be selected for charging if the system is overloaded with them. The proposed Gated Recurrent Unit (GRU) detector can detect lying electric vehicles with high accuracy (ACC) and a low false alarm rate. The GRU detector outperforms a Multilayer Perceptron (MLP) detector, and the MLP detector demonstrates a strong ability to detect new attacks that were not represented in the training dataset.

#### Research implications

While not a dedicated section, the paper points out that the dataset created can also be used for other applications, such as energy demand prediction for energy management applications. Simulation-based evaluation, synthetic attack data, limited attack scope, and dataset assumptions (Shafee et al., 2020). Trivedi et al. (2022) highlights a critical vulnerability in current electric vehicle charging coordination mechanisms and confirms that “lying” electric vehicles benefit significantly, receiving a higher chance of charging sooner. While transitioning from simulation to real-world implementation is a common progression in research, the provided source hints on issues with charging of scheduling that has been tough to resolve due to the complex nature of deep learning. Similarly, while the study’s accuracy for fault detection was 70%, there is need for future work to specifically aim for higher accuracy or to address the trade-off in computation time for data storage when using IPFS, despite these being identified as aspects of the system’s performance.

#### Practical implications

Shafee et al. (2020) primary focus was to create a dataset for training a detector to identify lying electric vehicles. Practitioners can utilize the dataset created for different applications including energy demand prediction for energy management applications. This suggests that the valuable dataset, comprising both honest and malicious electric vehicles charging data, has utility beyond the immediate scope of detecting false state of charge reports, offering a foundation for research in broader smart grid energy management. The main direction is improvement in cybersecurity of automated electric vehicle systems with the tool being deep learning resolutions to prevent compromises like lying electric vehicles. The study concludes that automated electric vehicles are a crucial component of smart factories, but they face significant challenges from cyberattacks and data loss that affect their monitoring and control. To address these challenges, the study successfully developed and demonstrated a hybrid IoT architecture which integrates model predictive control, dynamic programming, and deep neural network frameworks. This architecture is designed to detect and mitigate these threats effectively (Bergies et al., 2024; Basnet and Ali, 2020).

### Theme 3: electric vehicle societal integration

The electric vehicle’s societal integration has gone through multiple phases to achieve current results, i.e., energy efficiency and practicability, among others. A total of 88 articles has focused on the integration of electric vehicles in different communities. These articles highlight the reasons for pushing this endeavor, the implications, and the future goals in place to be achieved by this move. Under this theme, the focus is mainly on how energy is generated in both electric vehicles and grid, how safe electric vehicles can be made, and how costs of both maintaining the grid and sustaining electric vehicles. Furthermore, there is a focus on operational efficiency, battery hardware management, and state of charge estimation of batteries in electric vehicles.

Of the 88 articles, 73 articles (e.g., Zhang et al., 2022; Sun et al., 2023; Kosuru and Kavasseri Venkitaraman, 2023; Malek et al., 2021; Jahromi et al., 2024; Liu et al., 2025; Pei et al., 2019; Petkevicius et al., 2021; Ma et al., 2022; Koohfar et al., 2023; Kwon et al., 2025; Boulakhbar et al., 2022; Pan et al., 2023; Luo et al., 2021; Shahbazian and Guerriero, 2024; Shanmuganathan et al., 2022; Sheeba et al., 2022; Wu et al., 2024; Wei et al., 2022; Xia et al., 2025; Zhao et al., 2022) point out the energy management of electric vehicles and how it has been used to increase the number of electric vehicle users in the respective communities focused on in each article. A key outcome was how to prevent battery exhaustion in delay scenarios (like traffic jams), which is a simple estimation that could fail to account for (Laroui et al., 2019).

Another focal point is the state of charge estimation in six articles (e.g., Zhang et al., 2022; Sulaiman and Mir, 2024; Lin et al., 2022; Saba et al., 2023) with the use of deep learning as a tool to customize the energy management experience based on electric vehicle use by the owner (Zhang et al., 2022).

One article focuses on the Machine Theory of Mind (MTOM) paradigm used to model EV users’ charging behavior (Hu S. et al., 2021). In another article, there is a focus on how to improve the economic benefits of the distribution network by minimizing operating costs of using electric vehicles as day-to-day transportation options (Duan et al., 2023). Improved predictive control, validated through a high-accuracy simulation model and comparison against benchmark strategies under various driving conditions, is used to improve electric vehicle safety (Renold and Kathayat, 2024).

Fifty-two articles (e.g., Eddine and Shen, 2022; Kaplan et al., 2021; Pourvaziri et al., 2024; Pei et al., 2019; Basnet and Ali, 2020; Ma et al., 2022; Zhou et al., 2022; Sulaiman and Mir, 2024; Lin et al., 2022) focused on the operational efficiency of electric vehicles and the implementation of different technological advancements like deep learning to improve their reliability and use of less energy at a time. A central theme is the development of a novel solution to the problem of substantial uncertainty and sharp variations in electric load profiles introduced by PEV charging demands (Jahangir et al., 2020), optimizing Data Management and Scalability (Trivedi et al., 2022), and significant Improvement in Vehicle Motion Control Performance using various technologies like Brain-Inspired System Design, Unscented Kalman Predictor among others (Li et al., 2018).

In addition, 42 articles (e.g., Shafee et al., 2020; Pei et al., 2019; Ma et al., 2022; Sedhom et al., 2025; Salunkhe et al., 2022; Ha et al., 2021; Jahromi et al., 2024; Kaplan et al., 2021) look at grid integration of electric vehicles without energy surges. One study highlights that the increasing adoption of electric vehicles presents a significant impact on the electricity grid due to the inherent uncertainty of electric vehicle charging loads (Rasheed et al., 2023), Technology simplicity, LSTM can achieve good forecasting performance by utilizing fewer, carefully chosen features (Vishnu et al., 2023), and Leveraging Bayesian Deep Learning (BDL) for Probabilistic Forecasting to prevent abrupt energy surges (Zhou et al., 2022).

Lastly, 17 articles (e.g., Simsek et al., 2024; Li et al., 2024; Liu et al., 2025; Fallah et al., 2024; Khalatbarisoltani et al., 2024; Lee and Choi, 2024; Jose et al., 2025) investigate the battery hardware management issue as it influences energy utilization in electric vehicles. The study champions DRL, particularly the DDPG algorithm, as a powerful tool for electric vehicle battery management (Saba et al., 2023), Using LiFePO4 batteries, deep learning, transfer learning, and different battery management systems to improve battery preservation (Hu T. et al., 2021), Growing popularity of pure electric vehicles and the resultant surge in demand for charging infrastructure, particularly fast charging stations (Shah and Singh, 2023), and Ripple current prediction, deep learning, output current ripple, Bi-LSTM, CNN are essential for battery longevity and charging efficiency (Madumathi et al., 2024).

#### Exemplar studies

Exemplar studies under electric vehicle societal integration include two articles that highlight the different steps that have been taken to improve energy management in electric vehicles in general. This has been done mainly using the combination of both traditional and deep learning techniques that have been tested to achieve usable results. Alhazmi (2024) points out that research was carried out to focus on state of charge estimation, electric vehicle data collection, model optimization, and experimental setup and performance evaluation. The research uses experimental data from the CALCE (Center for Advanced Life Cycle Engineering) dataset provided by the University of Maryland. Raw time-series data from batteries are converted into State of Health-based data, and the Support Vector Machine-Recursive Feature Elimination algorithm is applied to pre-processed data. The paper introduces a hybrid model called the Diffusion Convolutional Recurrent Neural Network with a Support Vector Machine-Recursive Feature Elimination algorithm. The model’s adaptability and performance are explored using the CALCE dataset, which contains time-series data including parameters like voltage, current, temperature, and charge/discharge cycles which provides a variety to choose from. With this rigorous research process, there was enhanced safety and optimal Functioning of electric vehicles, development of robust battery management systems, improved battery lifespan prediction and energy management, contribution to deep learning and machine learning in battery technology, efficiency and interpretability in model design, addressing environmental and energy concerns, and a foundation for future research.

In addition, Alansari et al. (2024) also looks at the optimal placement of Electric Vehicle charging infrastructures, specifically Charging Stations and Dynamic Wireless Charging infrastructure, be determined for long-term planning, considering factors such as projected population growth, EV adoption forecasting, and consumer needs, with the objective of minimizing governmental expenditure on their construction. Dubai, UAE, was chosen as the case study due to the active role of the Dubai Electricity & Water Authority in promoting EV adoption and the Roads and Transport Authority’s initiatives, including the “EV Green Charger Project” and testing DWC infrastructure. The paper introduces a novel hybrid deep learning model, named SACGX, designed to accurately predict future variables essential for infrastructure planning and a Genetic Algorithm is employed to solve the mixed-integer nonlinear programming problem, justified by its prevalent use in similar optimal allocation problems. The paper focuses on the optimal long-term placement of electric vehicle charging infrastructures.

#### Research implications

The primary areas for future work identified within the different resources revolve around enhancing the performance of the proposed deep learning technologies, largely by improving the accuracy of future driving condition predictions, especially in dynamic real-world scenarios, and managing the inherent trade-offs in the different designs. This could be backed up by the proposed, for example, hybrid RNN-GRU deep learning approach as an effective and promising method for accurately predicting the state of electric vehicles, particularly mileage, contributing to better charging control, management, and overall reliability of electric vehicle operations. Another implication could be finding a way to combine different types of technologies to form better and simpler technology, i.e., hybrid technology, to solve multiple energy management challenges at the same time. This would in the long run be less expensive and will promote collaboration among different innovators. Further, improving data availability, quality, utilization, and addressing on-board computing limitations, i.e., better processing power for EVs to match GPUs and TPUs used in deep learning training. This would be a steppingstone to explore the integration of new optimization algorithms based on the pros and cons of the current ones, like the SACGX model (Alansari et al., 2024; Eddine and Shen, 2022; Rasheed et al., 2023; Petkevicius et al., 2021).

#### Practical implications

The first study emphasizes that integrating an accurate deep learning-based prediction model (LSTM) into an MPC framework is a promising approach for developing effective, robust, and near-optimal real-time energy management strategies for PHEVs, offering significant improvements in fuel efficiency compared to conventional rule-based methods (Sun et al., 2023). In addition, the paper proposes an Energy Management Strategy based on deep learning and improved model predictive control. Another study identifies a challenge in the Recursive Neural Networks (RNNs) feature extraction framework. The sources mention that the prediction outcomes from the GRU system “do experience some oscillations and errors.” In the quantitative comparison using Mean Square Error (MSE) and Mean Absolute Error (MAE) metrics, the LSTM model achieved slightly lower values (MSE 0.80, MAE 0.72) compared to the proposed RNN-GRU model (MSE 0.92, MAE 0.70) (Venkitaraman and Kosuru, 2023). Another article points out that State of Charge estimation using deep learning is an intersection of computer science, data science, and battery chemistry, fields that still present many complex problems. While deep learning is efficient, it does not provide a deeper understanding of battery state parameter changes from a chemical perspective. The authors envision deep learning as a promising technique for real-time battery management in the future, particularly with the expansion of computer science, advancements in data storage technology (like cloud storage), and the availability of high-quality data (Zhang et al., 2022).

Furthermore, macro-level societal acceptance and urban mobility integration remain heavily tethered to mitigating individual range anxiety and optimizing local distribution networks. Recent empirical data demonstrates that deploying machine learning architectures for intelligent brake-blending can significantly optimize vehicle-level energy reclamation in real-world urban driving environments (Milojević et al., 2026). Their findings reveal that transitioning from traditional rule-based mechanisms to a machine learning policy boosts regenerative energy recovery from a baseline of 30 to 50% under standard Urban Driving, and from 25 to 45% in dense, high-traffic Stop-and-Go cycles (Milojević et al., 2026). By utilizing data-driven control loops to reclaim up to half of a vehicle’s kinetic energy during daily commuting, these models directly lessen the cumulative frequency and urgency of grid-to-vehicle power draws. This systematically mitigates peak load stress on urban public charging networks, facilitating a more sustainable and seamless smart-city integration.

### Theme 4: implications of electric vehicle societal integration

After applying and emphasizing the use of electric vehicles in different communities, this theme, in 18 articles (e.g., Vishnu et al., 2023; Lin et al., 2022), aims to look at how the use of electric vehicles has impacted the lives of people, from renewable energy utilization, minimizing electric vehicle costs, to fulfilling Sustainable Development Goals. The main points looked at are the different ways the introduction of this new system of transportation and energy consumption has impacted the daily lives of users, researchers, and other stakeholders in this sector.

In general, the selected articles (e.g., Sotoudeh and Hom Chaudhuri, 2023; Ma et al., 2022; Sedhom et al., 2025; Jahromi et al., 2024; Alhazmi, 2024; Priyadarshini et al., 2025; Simsek et al., 2024; Dorokhova et al., 2021; Sundaramoorthi and Chitraselvi, 2024; Han et al., 2019) look at how renewable energy has been used over time after the introduction of electric vehicles on track to achieve carbon neutrality fulfilling sustainable development goals as advised by the United Nations. In one article, Ha et al. (2021) briefly looked at the potential policies in place and policy makers that could play a role in building or breaking the electric vehicle industry.

#### Exemplar studies

Sotoudeh and Hom Chaudhuri (2023) focused on the development of a novel learning-based hierarchical control framework that can jointly (simultaneously) optimize the vehicle’s driving cycle (velocity profile) and its powertrain’s power split (powertrain energy management) in a computationally tractable manner. This resulted in jointly optimizing the hybrid electric vehicles’ driving cycle (velocity profile) and its powertrain power split (powertrain energy management). It developed a novel framework that is computationally tractable and suitable for real-time implementation in vehicles, which often has limited computational power. To achieve this, the study proposes a novel learning-based hierarchical control framework that harnesses the benefits of both long- and short-term decision-making. The use of a Deep Neural Network (DNN) to learn and approximate complex control laws, thereby reducing online computational needs. The development of a Quadratic Programming-based projection algorithm to modify the DNN’s approximate optimal control solutions and ensure the satisfaction of system constraints was also a big breakthrough.

Ma et al. (2022), looked at how deep reinforcement learning and blockchain technology are used for optimal scheduling of electric vehicles in renewable energy-oriented power systems, is to review and summarize the application of state-of-the-art technologies for the optimal scheduling of electric vehicles within power systems that are increasingly integrating renewable energy. Deep Learning is primarily used for scenario generation, EV load prediction, flexibility prediction (adjustable power of EVs), and strategy optimization. DRL is primarily applied to the optimization of EV charging and discharging, and Blockchain addresses problems such as poor expansibility, lack of anonymity, and privacy in centralized EV energy management models. It aims to create a transparent, secure, and efficient trading model.

#### Research implications

A key direction for future research is obtaining large amounts of real data to enable a wider range of deep learning applications and integrating deep learning with big data is suggested as a future research area for its application in electric vehicle optimal scheduling. Deep learning can be applied to characterize and represent the uncertain behavior of electric vehicle users, which traditional methods struggle with, due to their powerful knowledge of extraction capabilities. Future work should focus on enhancing the convergence of DRL algorithms, especially complex problems, as this is a significant factor currently limiting its application in electric vehicle optimal scheduling. Realizing efficient hyperparameter searches is an effective way to improve the application prospects of Deep Reinforcement Learning. With the emergence of carbon markets, future EV optimal scheduling problems should not only consider the relationship between electric energy and traffic but also the impact of carbon emissions, for which DRL is considered an effective method (Ma et al., 2022; Eddine and Shen, 2022; Renold and Kathayat, 2024; Kaplan et al., 2021; Jahangir et al., 2020; Wang et al., 2023).

#### Practical implications

The proposed framework is effective in generating real-time applicable energy-efficient solutions for unseen driving cycles that share the same traffic pattern as deep neural network (DNN) training data. The use of a DNN-based Model Predictive Control (MPC) controller, which approximates the complex control law, substantially reduces the online computational needs compared to the baseline MPC controller. The significant reduction in online computation times did not come at the cost of a noticeable change in the controller’s performance in terms of fuel efficiency (measured in miles per gallon, mpg). The study reinforces that the hierarchical control framework (combining long-term PSOC and short-term learning-based MPC) allows for joint optimization of the vehicle’s driving cycle and powertrain energy management in a computationally efficient and real-time applicable manner, while facilitating charge balance constraint satisfaction (Sotoudeh and Hom Chaudhuri, 2023). An effective fault diagnosis approach is crucial for lowering maintenance costs and increasing the availability of electric vehicle systems for optimal use (Kaplan et al., 2021).

### Theme 5: challenges and solutions with electric vehicles using deep learning

While applying deep learning to electric vehicles, the researchers have been able to achieve multiple goals in eighty-one articles (e.g., Douaidi et al., 2021; Raj and Sakthivel, 2024; Basnet and Ali, 2020; Lee and Choi, 2024; Kumar and Jain, 2024; Jinil and Reka, 2019), but they have also battled through various challenges and have proposed viable solutions. These have been solved using both traditional and deep learning technologies to improve the main issues of fault detection, cybersecurity, and grid integration that facilitate the transition to reliable electric transportation.

In addition, cybersecurity is another important factor that has been focused on by eight articles (e.g., Trivedi et al., 2022; Shafee et al., 2020; Bergies et al., 2024; Basnet and Ali, 2020; Salunkhe et al., 2022; Douaidi et al., 2021; Raj and Sakthivel, 2024; Lee and Choi, 2024) to highlight the different ways deep learning could be utilized to improve cybersecurity in electric vehicles.

Of the 81, 10 articles (e.g., Kaplan et al., 2021; Trivedi et al., 2022; Ma et al., 2022; Hosseini et al., 2024; Liang et al., 2024; Wongchai et al., 2023; Salunkhe et al., 2022; Deng et al., 2023) focus on fault diagnosis and error prevention using deep learning techniques.

Then, grid integration comes in as a major influence in the transition to electric transportation around the world. This is concentrated on 42 articles (e.g., Eddine and Shen, 2022; Kaplan et al., 2021; Wang et al., 2023; Vishnu et al., 2023; Ren et al., 2022; Pourvaziri et al., 2024; Shafee et al., 2020; Pei et al., 2019; Zhou et al., 2022; Sedhom et al., 2025) that point out different steps have been taken to improve the grid reliability for the electric vehicle transition. Grid integration involves energy management, real world operational efficiency,

#### Exemplar studies

The chosen exemplar studies are going to focus on fault diagnosis, cybersecurity, and Grid integration of electric vehicles, pointing to how the different systems have been compromised as well as strengthened for better use. First, the research question being addressed by Basnet and Ali (2020) revolves around developing and evaluating a deep learning-based Intrusion Detection System for Electric Vehicle Charging Stations (EVCS) to effectively detect and classify Denial of Service (DoS) attacks, improving cybersecurity. The study highlights that EVCS are increasingly vulnerable to cyberattacks due to their integration with open communication layers and reliance on wireless technologies for scheduling, authentication, and energy management. The paper’s methodology centers on applying these deep learning algorithms to the CICIDS 2018 DoS attack dataset, which is selected for its inclusion of modern-day DoS attacks relevant to the EVCS scenario. The ultimate focus is on evaluating the performance of the proposed DNN and LSTM models using metrics such as accuracy, precision, recall, and F1-score, and demonstrating that both achieve high detection accuracy (over 99%), with LSTM generally outperforming DNN.

In another article, Kaplan et al. (2021) demonstrates the detection of faults in an electromechanical conversion chain for conventional or autonomous EVs. The research specifically focuses on identifying and diagnosing electrical faults, such as open-circuit and short-circuit faults in induction motors. The central novelty of the study is the development of an architecture for a fault diagnosis model using the feature extraction technique. The core implication is the significant improvement in the accuracy and effectiveness of fault diagnosis in electric vehicles. Timely and accurate fault diagnosis is essential for ensuring efficient operation and extending the lifespan of vital EV components, particularly the three-phase induction motors. Accurate fault detection allows EVs to be operated more securely and reliably, preventing extreme interruptions and losses that simultaneous faults can cause. The study strongly validates the capability of deep learning, particularly LSTM networks, to handle complex, non-linear time-series data inherent in electric vehicle sensor readings (currents, voltages, speeds).

Lastly, Rasheed et al. (2023) highlights that the core investigative method is the proposal and evaluation of an Improved Supervised Learning (ISL)-based Deep Neural Network (DNN) for accurately forecasting the load demand of EVs. The research incorporates and tests several DL architectures: Gated Recurrent Unit (GRU), Long Short-Term Memory (LSTM), Recurrent Neural Network (RNN), Fully Connected (FC), and Convolutional Neural Network (CNN). Real-world Dataset: The investigation utilizes a real-world EV charging dataset from Boulder City, USA. The raw data is pre-processed by splitting it into different time intervals to test various scenarios. Min-max scaling is applied to normalize the dataset, ensuring all features are on the same scale (between 0 and 1). After normalization, the dataset is divided, with 70% used as a training set to train the models and 30% as a testing set to predict the EV load. The ISL technique is applied through feature engineering, where important input features are extracted to create a new dataset.

#### Research implications

Under fault detection, the paper explicitly points to the implementation of the proposed LSTM-based fault diagnosis approach for Vehicle-to-Grid (V2G) technology in the context of a smart city. It is highly recommended that the fault diagnosis approach proposed in this paper, which is based on the Long Short-Term Memory (LSTM) network, is implemented for (V2G) technology in the context of a smart city. This recommendation implies extending the current research beyond internal electric vehicle (EV) fault diagnosis to its broader integration within smart energy infrastructures, where EVs can interact with the grid (Kaplan et al., 2021). Future research could delve into the significant impact of high-capacity charging stations on the magnitude and direction of short-circuit currents within distribution networks. The authors suggest integrating fault detection processes with protection coordination and the management of electric vehicle charging and discharging which will strengthen the reliability rate of the grid. Exploring the application of more advanced deep learning models is recommended. Specifically, Graph Neural Networks and sequence prediction models like Transformers are mentioned as potentially playing a critical role in processing complex, large-scale network data, especially in scenarios with high distributed generation unit and energy storage penetration (Hosseini et al., 2024).

Further, Rasheed et al. (2023) highlights that the accurate electric vehicle load demand predictions, facilitated by the proposed Improved Supervised Learning (ISL) technique, enable stakeholders to optimize the allocation of resources for EV charging infrastructure. With a more precise understanding of future charging loads, the study models allow for effective planning of infrastructure capacity. The research itself contributes to advancing load forecasting models for EVs by leveraging deep learning architectures and incorporating the novel ISL technique. The ISL technique proves beneficial for handling nonlinearities, incorporating multivariate inputs, and learning from big data. Looking to the future, the study lays groundwork for developing smart charging strategies.

#### Practical implications

The study focus on fault detection in electric vehicles has demonstrated efficacy and high accuracy of the LSTM deep learning network for diagnosing electrical faults in the electromechanical conversion chain of electric vehicles, significantly outperforming other machine learning techniques like ANNs. This is largely attributed to Long Short-Term Memory’s (LSTM) ability to handle time-series data and capture long-term dependencies through its memory cells (Kaplan et al., 2021). This can be applied to new technologies in the electric vehicle sector to incorporate machine learning as a tool for ease of recognition of faults based on electric vehicle data patterns.

One paper outlines the development and evaluation of a novel deep learning-based Intrusion Detection System for Electric Vehicle Charging Stations (EVCS) to detect Denial Service attacks (DoS). The study compared Deep Neural Network (DNN) and Long Short-Term Memory (LSTM) algorithms, finding that the proposed LSTM-based IDS significantly outperformed the DNN-based IDS. LSTM demonstrated superior performance in terms of accuracy, precision, recall, and F1-score for both binary and multiclass classification. Crucially, the LSTM model achieved a desired training accuracy of approximately 99.95% within just 10 epochs, significantly faster than the DNN’s 70 epochs. This led the authors to conclude that LSTM is an effective algorithm for detecting DoS attacks in EVCS, with the potential to safeguard against these critical infrastructures boosting cybersecurity. The model is also designed for scalability and interoperability, allowing it to detect new attacks by incorporating additional data (Basnet and Ali, 2020).

Further, Rasheed et al. (2023) highlights that the increasing adoption of electric vehicles (EV) presents a significant impact on the electricity grid, primarily due to the inherent uncertainty of EV charging loads. This is motivated by the need for novel EVLF methods based on deep learning models, and the core innovation is the proposal of an ISL-based Deep Neural Network (DNN) for forecasting EV load demand. The overarching purpose and implication of this improved accuracy form a crucial part of grid stability. The accurate load demand predictions facilitated by the ISL-enhanced models also have significant implications for the planning and management of EV charging stations.

A prominent operational challenge in wide-scale EV fleet deployment involves managing maintenance overheads and predicting system degradation without relying on computationally prohibitive, mathematically intensive structural formulations. To resolve this, recent literature demonstrates a paradigm shift toward neural network-driven forecasting within performance-based logistics systems. As established in (Dejanović et al., 2025), the deployment of fully connected neural networks and long short-term memory networks enables exact optimization of component repair rates without the need for complex, explicit mathematical derivations. This data-driven optimization directly tackles the challenge of fleet resource constraints, providing logistics managers with a scalable mechanism to dynamically project asset availability and minimize unexpected vehicle downtime based on historical failure data (Dejanović et al., 2025).

Simultaneously, navigating complex, non-linear safety subsystems during abrupt decelerations represents an ongoing control bottleneck, where balancing emergency wheel-slip safety with energy conservation is highly volatile. Machine learning models provide a robust alternative to classical stochastic controllers by precisely modulating friction and regenerative dynamics. Empirical testing indicates that a machine learning-based brake blending policy pushes highway energy recuperation rates from a baseline of 40 to 60% and notably recovers up to 25% of energy during critical Emergency Braking conditions compared to a mere 10% baseline (Milojević et al., 2026). Among predictive frameworks, random forest algorithms excel in resolving these highly non-linear safety spaces, yielding the minimum root mean squared error when mapping stopping trajectories and mitigating brake-pad wear. This demonstrates that targeted machine learning applications can successfully eliminate historic engineering trade-offs between physical component preservation, driving safety, and energy efficiency.

## Discussion and conclusion

This study advances prior reviews by moving beyond descriptive reporting to provide a structured analytical comparison of deep learning approaches, highlighting methodological patterns, performance trends, and critical gaps in evaluation practices. The findings of this review should be interpreted as a pattern-based and exploratory synthesis of the literature rather than a comparative evaluation of study quality or effectiveness. Due to the absence of a formal quality assessment framework and the diversity of study designs included, the conclusions reflect recurring trends and thematic patterns across studies rather than weighted judgments based on methodological rigor. As such, the results are intended to map the research landscape and identify emerging directions, rather than to establish evidence of hierarchies or definitive performance comparisons.

Although the five themes in the findings section are distinct, several papers in this SLR looked at intersections across themes. For example, Duan et al. (2023) looks at the intersection of software development and societal integration by directly tackling the problem of EV charging loads overlapping with conventional peak loads, which creates “peak curves” that challenge system operations, introduces a novel framework that formulates the dynamic scheduling problem of distribution networks as a Markov Decision Process, enhancing the economic benefits of the distribution network and reduce its operating costs, and data-driven approach, where the DDPG algorithm is trained offline using historical load and photovoltaic output data. Zegong et al. (2022) focuses on developing and validating an optimal energy management strategy for Hybrid Electric Vehicles using offline Deep Reinforcement Learning. This approach was specifically chosen to overcome the inherent disadvantages of online DRL algorithms, such as their high trial-and-error costs, demanding computational requirements for controllers, and difficulties with simulation-to-reality transfer in real-life applications. Lastly, Alansari et al. (2024) focus on the optimal long-term placement of Electric Vehicle (EV) charging infrastructures. Specifically, it addresses the strategic allocation of two types of charging facilities: Charging Stations and Dynamic Wireless Charging (DWC) infrastructure. Minimizing governmental expenditure on the construction of both CS and DWC infrastructure. Meeting the needs of mass-market EV consumers, ensuring adequate charging infrastructure availability. Long-term planning for infrastructure development.

Our descriptive analysis of articles further highlighted the ability to test and apply existing technologies to new research on electric vehicle usage and propose new frameworks for better user experience. In addition, studies concentrating on fundamental research were more likely than those focused on electric vehicle technology development to use advanced research methods and more likely than those focused on electric vehicle technology improvements to use larger sample sizes over multiple countries and institutions. As the community creates knowledge around electric vehicles while they are getting used to them more often, these findings suggest a step to encourage the adoption of electric vehicles as a reliable transportation means, given their simplicity and predictability. Given the large number of research papers focused on deep learning, this indicates the need for other technologies applicable to electric vehicles to get a platform for research exposure for the development of a diverse range of resources to utilize, which fuels growth.

In addition, examination of the intersections of themes identified in this SLR generates additional observations and implications for research and practice. For example, a potential research question could be: What impact has the development and implementation of deep learning in the electric vehicle industry had on improving grid integration, operational efficiency, and electric vehicle data analysis and collection, and how has rapid software advancement played a role in accelerating this move? Studies could also be conducted to better understand the challenges related to deploying a certain amount of technology into electric vehicles as they relate to real-world usage by consumers with different driving behaviors. Finally, continuously examining the relationship between feedback and assessment in evaluating online student outcomes is another potential direction for further research, motivated by the intersection of identified themes.

Several studies demonstrate the practical application of deep learning in real-world electric vehicle systems. For example, LSTM-based models are widely used for state-of-charge and battery health prediction using operational battery datasets, enabling more accurate energy management. Deep learning techniques such as CNN and LSTM have also been applied for fault detection and diagnostics using real-time sensor data, improving system reliability, and safety. In addition, deep reinforcement learning approaches are increasingly used for optimizing charging strategies and energy management in dynamic environments. Real-world applications also extend to charging infrastructure planning, where data-driven models are used to determine optimal placement of charging stations based on usage patterns and demand forecasting. These examples highlight the growing transition of deep learning applications from simulation-based studies to real-world implementation in electric vehicle systems.

Future industrial applications of deep learning in the EV sector are poised to transition from simulation-based research to real-time, integrated production systems. A critical direction involves enhancing on-board computing power to match external processing capabilities, enabling deep learning models to function within real-time battery management systems (Zhang et al., 2022). This evolution will allow for the deployment of near-optimal energy management strategies in plug-in hybrids by integrating LSTM networks into Model Predictive Control frameworks (Mei et al., 2023). Furthermore, the industry is moving toward active grid management. Future systems will likely combine load forecasting with control algorithms to facilitate smart charging strategies that account for grid limitations and user preferences. This includes scaling predictive capabilities from single stations to regional demand forecasting and implementing fault diagnosis within Vehicle-to-Grid infrastructures in smart cities. In terms of vehicle safety, low-cost Advanced Driver Assistance Systems can be integrated into existing vehicle architectures to identify driver behavior and optimize safety layers without substantial hardware changes (Balan et al., 2022). To increase reliability, the industrial adoption of Digital Twin technology and Explainable AI will address current limitations in model interpretability and battery health prediction. Finally, the development of reinforcement learning algorithms will enable vehicles to autonomously adjust parameters in real time to balance driving efficiency with passenger comfort (Laroui et al., 2019).

Despite the growing application of deep learning in electric vehicles, several limitations persist, including data dependency, lack of standardized benchmarking, reliance on simulation-based datasets, computational complexity, and limited model interpretability. These challenges highlight the need for more robust datasets, consistent evaluation frameworks, and scalable model designs to improve real-world applicability.

In conclusion, the SLR features articles published between 2015 and 2025 on the applications of deep learning in the electric vehicle industry. A total of 683 articles were retrieved in total from seven databases using one search term, “electric vehicles + deep learning,” and 121 of them summarized their themes and publication trends. Across the 121 articles, we identified guidelines for improving the experience of using electric vehicles and main transportation means including (1) improving the electric vehicle charging infrastructure, battery hardware management, minimizing EV costs, and renewable energy utilization, (2) enhancing the electric vehicle safety control systems, cybersecurity, and new software development and implementation, (3) expounding on model optimization, design evaluation, and operational efficiency, and (4) fulfilling the sustainable development goals, environmental planning, and EV energy management, and (5) easing fault diagnosis, EV data handling and utilization, assessing human driving behavior, and EV policy analysis. Through the analysis of the different trends, we determined the research on electric vehicles and deep learning, applications, challenges, and solutions to be extremely relevant. We also realized the need for more theory-based research conducted at scale and focused on spreading different perspectives on the interpretation of applications of different technologies in the electric vehicle industry today. In summary, the expansion of this review to integrate state-of-the-art 2025 and 2026 frameworks underscores a critical technological evolution: deep learning is migrating rapidly from isolated virtual simulations to real-world mechanical and logistical deployments. The synthesized literature reveals that advanced neural architectures (such as FCNNs and LSTMs) can successfully decouple fleet logistical optimization from tedious mathematical equations (Dejanović et al., 2025), while machine learning models (such as Random Forest) resolve highly non-linear vehicle dynamics to dramatically elevate regenerative efficiency across diverse urban and emergency driving cycles (Milojević et al., 2026). Addressing these multi-domain adjustments confirms that future EV scalability relies on deep learning ability to concurrently resolve micro-level mechanical safety boundaries and macro-level logistics infrastructure.

## Data Availability

The original contributions presented in the study are included in the article/Supplementary material, further inquiries can be directed to the corresponding author/s.
