# Supplementary material for: A systematic literature review exploring the application of deep learning in electric vehicles from 2015 to 2025
Source: Front Artif Intell. 2026 Aug 11;9:1826633. doi: 10.3389/frai.2026.1826633 (PMC13503603; doi:10.3389/frai.2026.1826633)
Supplement: Supplementary file 1 [file Table_1.docx]

**APPENDIX A:** CODES, CODE DESCRIPTIONS, AND EXEMPLARS

| **CODE** | **DESCRIPTION** | **EXEMPLAR** |
| --- | --- | --- |
| EV data handling | Topics related to research data being collected in deep learning and electric vehicles. | “The study demonstrated that integrating the proprioceptive system with a yaw rate tracking controller, specifically Second-Order Sliding Mode Control (SOSMC), significantly mitigated performance degradation caused by communication delays and measurement noises.” ([Li et al. 2018](https://ieeexplore.ieee.org/abstract/document/8411139/?casa_token=NClc6f8qhaoAAAAA:j75Qadnpi4fY52y4UH9kkdYx1OJNFSNUX5WqxmbiuPWniHP11FCJPf5ifb8ePlViD0lqMGjjuw)) |
| EV data utilization | Topics related to research data being utilized in the direction of electric vehicles and deep learning. | “The study indicates that while the primary focus was to create a dataset for training a detector to identify lying Electric Vehicles (EVs), the created dataset can also be utilized for other applications, such as energy demand prediction for energy management applications” ([Shafee et al. 2020](https://ieeexplore.ieee.org/abstract/document/9210521)). |
| EV energy management | Topics related to electric vehicle battery energy being utilized efficiently for maximum range. | “The paper introduces a novel deep learning-based approach to tackle charging demand prediction. Temporal Encoder-Decoder + LSTM (T-LSTM-Enc): This component pre-trains the data to extract hidden relationships. Temporal LSTM (T-LSTM-Ori-Time Features) Concatenated: This part is designed to capture the impact of time features on charging data” ([Pei et al. 2019](https://link.springer.com/article/10.1007/s11431-018-9396-0)). |
| Grid integration | Topics related to how electric vehicles are impacting the electricity grid and how the energy management systems are adjusting to incorporate the use of electric vehicles as transportation means without causing energy surges. | “The paper finds that a sophisticated deep learning architecture specifically designed to capture various temporal dependencies and incorporate time-series features, built upon processed and analyzed charging data (including periodicity classification), yields the most accurate predictions for both individual EV charging stations and the overall charging demand of the system.” ([Eddie & Shen 2022](https://link.springer.com/article/10.1007/s11227-022-04428-0)). |
| Human driving behavior | Topics related to human behavior while driving. How electric vehicles drivers make decisions, and these patterns are learned by electric vehicles using deep learning. | “The core of the paper's focus is on integrating the Machine Theory of Mind (MTOM) paradigm into the forecasting model. This integration aims to consider two crucial factors that influence EV charging demand: The user's living habits, which refer to the fixed weekly plug-in time habits of EV users. The short-term trend of charging demand variation, reflecting the current short-term changes in demand” ([Hu et al. 2021](https://ieeexplore.ieee.org/abstract/document/9490147?casa_token=NeUtwM8VREgAAAAA:uAObW4hReFRd9_MM1a62wfAJkO3ZHkFkH5XuijZek8XhXRlugQnZ3whSEZHPwhrTWHUK283s_A)). |
| EV safety measures | Topics related to how to make electric vehicles safer to use. This links to mastering driving patterns and using deep learning to master road nature and optimize safety. | “Enhances vehicle safety and stability during critical steering maneuvers; Reduces reliance on empirical tire force models, simplifying control system design; Demonstrates the potential of deep reinforcement learning for real-time vehicle control systems” ([Wei et al. 2022](https://ietresearch.onlinelibrary.wiley.com/doi/10.1049/itr2.12176)). |
| Fault diagnosis | Topics related to making electric vehicles more efficient and easier to fix. This could also be in line with reducing faults by preventive methods of improving vehicle efficiency. | “The deep learning models, specifically CNN and LSTM, were effective in predicting the presence of faulty data in EVs. For air tire pressure fault, the CNN model achieved a prediction accuracy of 70%. The simulation results showed a correlation between prediction accuracy on train and test datasets, and a loss curve for training and testing datasets was also depicted. For temperature (thermal) fault anomaly detection, the LSTM model also achieved a prediction accuracy of 70%. For battery fault detection, which involved predicting the State of Health using an LSTM model, the framework also achieved a prediction accuracy of 70%.” ([Trivedi et al. 2022](https://www.mdpi.com/2227-7390/10/19/3626)). |
| Operational efficiency | Topics related to electric vehicles being efficient in general with energy use and making them a better option unlike other transportation means. | “The DDPG-based method not only achieves high accuracy but also demonstrates less convergence time and reduces the overall driving cost compared to existing state-of-the-art models for EV batteries. The study's findings show that the proposed DDPG-based approach outperforms baseline methods in terms of both accuracy and robustness  ” ([Saba et al. 2023](https://ietresearch.onlinelibrary.wiley.com/doi/10.1049/stg2.12110)). |
| Forecasting accuracy | Topics related to predicting actions of drivers and traffic behaviors. This would promote safety and improve energy management through consistent patterns learnt using deep learning. | “The proposed hybrid classification task, which combines K-means (unsupervised) and a deep Long Short-Term Memory (LSTM) network (supervised), successfully autonomously discerns hidden travel behavior patterns within historical PEV data. Allocating a specific deep LSTM forecasting network to each identified travel pattern cluster (referred to as C-Deep) significantly improves forecasting accuracy compared to using a single forecasting network for all unclassified data (UC-Deep)” ([Jahangir et al. 2020](https://ieeexplore.ieee.org/abstract/document/9102300/?casa_token=TYT8pzRjduwAAAAA:30JJQ98ZlkXsfK6rE04M918nebW1Va0vO2d5vE0oKNHJPBwuB5XI5y3C_XYXl2oZGpC3FyMIfQ)). |
| Cybersecurity | Topics related to data protection of electric vehicles to prevent corruption and errors during execution. | “There is a significant technical gap in cybersecurity research for Electric Vehicle Charging Stations, noting that very limited work has been done in modelling and proposing strategies to deal with cyberattacks in this domain. Proposed Deep Learning-Based Intrusion Detection System (IDS): The study proposes a novel deep learning-based IDS to detect and classify DoS attacks in EVCS environments” (B[asnet & Ali 2020](https://ieeexplore.ieee.org/abstract/document/9243152/?casa_token=BhLMz3marQQAAAAA:RWOOHqv7SgYS5Ok11rS864lHW-C4kNLxk-6PhGWb4BjuWYXTvbgJ8fj35aHFZgAt0uEd3Ops7g)). |
| Fulfilling SDGs (Sustainable Development Goals) | Topics related to protecting the environment is even better in relation to using renewable energy and reducing pollutants. | “The research can be considered a primary step toward designing an efficient, coordinated EV load scheduling and management system. Contribution to Sustainable Development Goals (SDGs): The work aligns with the United Nation's Sustainable Development Goals (SDGs), specifically Goal 7 (Affordable and Clean Energy), Goal 11 (Sustainable Cities and Communities), and Goal 12 (Responsible Consumption and Production)” ([Vishnu et al. 2023](https://www.mdpi.com/2032-6653/14/9/266)). |
| EV software optimal capabilities | Topics related to the limits of software used by electric vehicles. This expounds on how deep learning could be used and what limits should be looked at without impacting performance and reliability. | “Several control strategies are integrated into comprehensive BTM control systems to ensure battery safety, extend battery life, and optimize EV efficiency. These include Temperature Control, Energy Management, Fault Detection and Diagnostics, Fast Charging Control, State of Charge (SoC) and State of Health Control, Thermal Runaway Prevention, and Regenerative Braking Control” ([Ali et al. 2024](https://www.sciencedirect.com/science/article/pii/S2090447924002831)) |
| Design evaluation | Topics related to how electric vehicles are designed, specifically to improve efficiency and facilitate operational efficiency. | “The study demonstrated that integrating the proprioceptive system with a yaw rate tracking controller, specifically Second Order Sliding Mode Control (SOSMC), significantly mitigated performance degradation caused by communication delays and measurement noises” ([Lin et al. 2018](https://ieeexplore.ieee.org/abstract/document/8411139/?casa_token=NClc6f8qhaoAAAAA:j75Qadnpi4fY52y4UH9kkdYx1OJNFSNUX5WqxmbiuPWniHP11FCJPf5ifb8ePlViD0lqMGjjuw)). |
| Renewable energy utilization | Topics related to maximizing the use of renewable energy sources rather than reducing pollution and making transportation more efficient in terms of energy usage. This focuses on using energy that can be easily recreated without waste. | “The study confirms the DDPG strategy's suitability for solving the dynamic economic dispatch problem in distribution networks that integrate renewable energy and electric vehicles. The work was supported by the State Grid Technology Project from the State Grid Corporation of China, suggesting its potential practical application and relevance to large-scale power grid operations” ([Y. Duan et al. 2023](https://ieeexplore.ieee.org/document/10166611/)) |
| Model optimization | Topics related to how newer electric vehicle models could be optimally made better compared to older ones to prevent redundancy. | “The high-precision forecasting provided by the LSTLNet with the Smooth Band Limit Maximum Likelihood (SBLM) estimator is crucial for Residential Electric Vehicle (REV) aggregators” ([Jahromi et al. 2024](https://ietresearch.onlinelibrary.wiley.com/doi/full/10.1049/gtd2.13276)) |
| Minimizing EV costs | Topics related to reducing how much it costs to operate electric vehicles, making them the cheapest transportation alternative. | “Accurately forecasting Electric Vehicle (EV) charging demand is crucial for increasing utilization for service companies, reducing costs for both companies and car owners, and alleviating the burden on electric grid stations” ([Eddine & Shen 2022](https://idp.springer.com/authorize/casa?redirect_uri=https://link.springer.com/article/10.1007/s11227-022-04428-0&casa_token=sixrMNMXKBMAAAAA:l4Xd9cll4EfQ-AQEa1CxdxeRCYOy8YoyCX_1FuRx_DEwENO5PF6PbJNwv0vI4-nEBqvc4tVXwZJza74Fdg)). |
| EV infrastructure improvements | Topics related to how electric vehicles have impacted the different infrastructure like roads and charging stations. | “The proposed framework, particularly through the use of the Hybrid Archimedes-Genetic algorithm for optimal Electric Vehicle Charging Station placement, significantly improves grid reliability” ([Sedhom et al. 2025](https://www.sciencedirect.com/science/article/pii/S0142061525000961)). |
| Battery hardware management | Topics related to how electric vehicle batteries are built and how they are designed for maximizing energy storage and longevity through transportation use. | “Optimized Battery Management Systems (BMS): The superior performance of the DLM in various management aspects—charging, capacity, terminal, and efficiency—compared to existing methods implies that deep learning can form the backbone of more intelligent and predictive BMS” ([S.Kumar Shah & M. Singh 2023](https://ieeexplore.ieee.org/document/10100037/)). |
| EV software development | Topics related to improving the software used in electric vehicles. This is directly linked to deep learning and how the new software is being used and improved with time to solve electric vehicle challenges being faced. | “Deep Reinforcement Learning (DDQN) Algorithm Implementation:  The study uses DDQN, an enhancement over the traditional Deep Q Network (DQN), to mitigate the problem of Q-value overestimation commonly found in DQN” ([Xia et al. 2025](https://www.mdpi.com/2032-6653/16/3/184)). |
| EV policy analysis | Topics related to policies and regulations but governing bodies regarding the use of electric vehicles, for example decision-making on policies that govern electric vehicle infrastructure placement. | “Enables real-time tracking and analysis of EV infrastructure issues for public policy and automates the extraction of consumer insights from unstructured data, improving decision-making for EV infrastructure management. Highlights underserved areas in EV infrastructure, especially in smaller micropolitan areas” ([Ha et al. 2021](https://www.sciencedirect.com/science/article/pii/S2666389920302658)). |

**APPENDIX B:** CLASSIFICATION OF REVIEWED STUDIES BASED ON SPECIFIC THEMES

| **AUTHORS** | **COUNTRY OF AFFILIATION OF FIRST AUTHOR** | **TITLE** |
| --- | --- | --- |
| Mohammed Laroui, Aicha Dridi, Hossam Afifi, Hassine Moungla, Michel Marot, Moussa Ali Cherif ([2019](https://ieeexplore.ieee.org/abstract/document/8766580/?casa_token=wvOAnWO2cYEAAAAA:aaxF1Z4hoU94QthZbf7imwU-lZqcx8a-QZ-91oH669Y0oH3dnxP07In7gmYeXKDXp8bksW4IbA)) | France | Energy Management For Electric Vehicles in Smart Cities: A Deep Learning Approach |
| Mekkaoui Djamel Eddine,  Yanming Shen  ([2022](https://doi.org/10.1007/s11227-022-04428-0)) | China | A deep learning based approach for predicting the demand of electric vehicle charge. |
| Gunapriya Balan, Singaravelan Arumugam, Suresh Muthusamy, Hitesh Panchal, Hossam Kotb, Mohit Bajaj, Sherif S. M. Ghoneim, Kitmo ([2022](https://doi.org/10.1155/2022/8548172)) | India | An Improved Deep Learning-Based Technique for Driver Detection and Driver Assistance in Electric Vehicles with Better Performance. |
| Ashwin Kavasseri Venkitaraman,Venkata Satya Rahul Kosuru ([2023](https://doi.org/10.24018/ejece.2023.7.1.485)) | USA | Hybrid Deep learning mechanism for charging control and management of electric vehicles. |
| A. Pravin Renold; Neeraj Singh Kathayat ([2024](https://ieeexplore.ieee.org/abstract/document/10477658/)) | India | Comprehensive review of machine learning, deep learning, and Digital Twin Data-Driven Approaches in Battery Health Prediction of Electric Vehicles. |
| Youssef Nait Malek, Mehdi Najib, Mohamed Bakhouya, Mohammed Essaaidi ([2021](https://ieeexplore.ieee.org/abstract/document/9321203/)) | Morocco | Multivariate deep learning approach for electric vehicle speed forecasting. |
| Ahmed A. Shafee, Mostafa M. Fouda, Mohamed M. E. A. Mahmoud, Abdulah Jeza Aljohani, Waleed Alasmary, Fathi Amsaad ([2020](https://ieeexplore.ieee.org/abstract/document/9210521/)) | USA | Detection of lying electrical vehicles in charging coordination using deep learning. |
| JiaZheng Pei, YiXin Su, DanHong Zhang, Yue Qi, ZhiWen Leng  ([2019](https://doi.org/10.1007/s11431-018-9396-0)) | China | Velocity forecasts using a combined deep learning model in hybrid electric vehicles with V2V and V2I communication. |
| Shimaa Bergies, Tawfiq M. Aljohani, Shun-Feng Su, Mahmoud Elsisi  ([2024](https://ieeexplore.ieee.org/abstract/document/10572004/?casa_token=aoe_ay6CyG4AAAAA:dT-RvIn5H_N2Hq8MmSyciCwiwC7cfFToaxmcUzFmiia_1t-MXn8FIhszT2nq9dzzsmtWpv-yag)) | Taiwan | An IoT-Based Deep-Learning architecture to secure automated electric vehicles against cyberattacks and data loss. |
| Manoj Basnet, Mohd. Hasan Ali ([2020](https://ieeexplore.ieee.org/abstract/document/9243152/?casa_token=BhLMz3marQQAAAAA:RWOOHqv7SgYS5Ok11rS864lHW-C4kNLxk-6PhGWb4BjuWYXTvbgJ8fj35aHFZgAt0uEd3Ops7g)) | USA | Deep learning-based intrusion detection system for electric vehicle charging station. |
| Nectar Jinil, Sofana Reka ([2019](https://ieeexplore.ieee.org/abstract/document/8719243/?casa_token=m27pOcuXpvIAAAAA:_2nsBWw901BQWvgqb_FJMkHKD4pY3kGOq1d1H3cYHOSkpFJ4qH3E-M4xnMaWvpzSr7rMkDBEhw)) | India | Deep Learning method to predict Electric Vehicle power requirements and optimizing power distribution. |
| [Linas Petkevicius](https://dl.acm.org/doi/10.1145/3469830.3470915), Simonas Saltenis, [Alminas Civilis](https://dl.acm.org/doi/10.1145/3469830.3470915), Kristian Torp ([2021](https://doi.org/10.1145/3469830.3470915)) | Lithuania | Probabilistic deep learning for Electric-Vehicle Energy-USE prediction. |
| Wenshuai Ma, Junjie Hu, Li Yao, Zhuoming Fu, Hugo Morais, Mattia Marinelli ([2022](https://doi.org/10.1049/enc2.12071)) | China | New technologies for optimal scheduling of electric vehicles in renewable energy‐oriented power systems: A review of deep learning, deep reinforcement learning and blockchain technology. |
| Dona George, Sivraj P ([2021](https://ieeexplore.ieee.org/abstract/document/9532912/?casa_token=nhkWd1GkVhIAAAAA:HYDcHH0kLeDLlvLspab0AI64TTT1B8HzG22FzA6Nu9hEeUlqD-JnyeKtj9mH14WG8lvRARS0Hg)) | India | Driving Range Estimation of Electric Vehicles using Deep Learning. |
| [Satish S. Salunkhe](https://link.springer.com/article/10.1007/s11227-021-04186-5#auth-Satish_S_-Salunkhe-Aff1), [Shelendra Pal](https://link.springer.com/article/10.1007/s11227-021-04186-5#auth-Shelendra-Pal-Aff2), [Abhishek Agrawal](https://link.springer.com/article/10.1007/s11227-021-04186-5#auth-Abhishek-Agrawal-Aff3), [Ravi Rai](https://link.springer.com/article/10.1007/s11227-021-04186-5#auth-Ravi-Rai-Aff4), [S. S. Sreeja Mole](https://link.springer.com/article/10.1007/s11227-021-04186-5#auth-S__S__Sreeja-Mole-Aff5) & [Bos Mathew Jos](https://link.springer.com/article/10.1007/s11227-021-04186-5#auth-Bos_Mathew-Jos-Aff6) ([2022](https://doi.org/10.1007/s11227-021-04186-5)) | India | Energy optimization for CAN bus and media controls in electric vehicles using deep learning algorithms. |
| Ali Jamali Jahromi, Mohammad Reza Masoudi, Mohammad Mohammadi, Shahabodin Afrasiabi ([2024](https://doi.org/10.1049/gtd2.13276)) | Iran | Improving electric vehicle charging forecasting: A hybrid deep learning approach for probabilistic predictions. |
| Ruikang Luo, Yicheng Zhang, Yao Zhou, Hailin Chen, Le Yang, Jianfei Yang ([2021](https://ieeexplore.ieee.org/abstract/document/9564633/)) | Singapore | Deep learning approach for long-term prediction of electric vehicle (EV) charging station availability. |
| [Mohamad Alansari](https://ietresearch.onlinelibrary.wiley.com/authored-by/Alansari/Mohamad), [Ameena Saad Al-Sumaiti](https://ietresearch.onlinelibrary.wiley.com/authored-by/Al%E2%80%90Sumaiti/Ameena+Saad), [Ahmed Abughali](https://ietresearch.onlinelibrary.wiley.com/authored-by/Abughali/Ahmed) ([2024](https://ieeexplore.ieee.org/abstract/document/9564633/)) | UAE | Optimal placement of electric vehicle charging infrastructures utilizing deep learning. |
| Tianyu Hu, Kailong Liu, Huimin Ma ([2021](https://ieeexplore.ieee.org/abstract/document/9490147/?casa_token=NeUtwM8VREgAAAAA:uAObW4hReFRd9_MM1a62wfAJkO3ZHkFkH5XuijZek8XhXRlugQnZ3whSEZHPwhrTWHUK283s_A)) | China | Probabilistic electric vehicle charging demand forecast based on deep learning and machine theory of mind. |
| [R. Sundaramoorthi,](https://link.springer.com/article/10.1007/s40998-024-00767-1#auth-R_-Sundaramoorthi-Aff1) [S. Chitraselvi](https://link.springer.com/article/10.1007/s40998-024-00767-1#auth-S_-Chitraselvi-Aff2)  ([2024](https://doi.org/10.1007/s40998-024-00767-1)) | India | Integration of renewable resources in electric vehicle charging management systems using deep learning for monitoring and optimization. |
| Ahmed Ihsan Simsek, Erdinç Koç, Beste Desticioglu Tasdemir, Ahmet Aksöz, Muammer Turkoglu, Abdulkadir Sengur ([2024](https://doi.org/10.3390/app142310974)) | Turkey | Deep Learning Forecasting Model for Market Demand of Electric Vehicles. |
| Juncheng Zhu, Zhile Yang, Monjur Mourshed, Yuanjun Guo, Yimin Zhou, Yan Chang, Yanjie Wei, Shengzhong Feng ([2019](https://doi.org/10.3390/en12142692)) | China | Electric Vehicle Charging Load Forecasting: A Comparative Study of Deep Learning Approaches. |
| Lian-ge He, Jun Song, Yan Zhang, Xin-yang Wu  ([2025](https://doi.org/10.1007/s11227-024-06748-9)) | China | Deep learning-based electric vehicle transmission system speed ratio prediction research. |
| Qun Wang, Fei Ju, WeiChao Zhuang, LiangMo Wang  ([2022](https://doi.org/10.1007/s11431-021-1994-7)) | China | Ecological cruising control of connected electric vehicle: a deep reinforcement learning approach. |
| Dan Zhou, Zhonghao Guo, Yuzhe Xie, Yuheng Hu, da Jiang, Yibin Feng, Dong Liu ([2022](https://doi.org/10.3390/en15176195)) | China | Using Bayesian Deep Learning for Electric Vehicle Charging Station Load Forecasting. |
| Jaikumar Shanmuganathan, Aruldoss Albert Victoire, Gobu Balraj, Amalraj Victoire  Amalraj Victoire ([2022](https://doi.org/10.3390/su141610207)) | India | Deep Learning LSTM Recurrent Neural Network Model for Prediction of Electric Vehicle Charging Demand. |
| Irum Saba, Muhammad Tariq, Mukhtar Ullah, H. Vincent Poor ([2023](https://doi.org/10.1049/stg2.12110)) | Pakistan | Deep reinforcement learning based state of charge estimation and management of electric vehicle batteries. |
| Juncheng Zhu, Zhile Yang, Yuanjun Guo, Jiankang Zhang, Huikun Yang ([2019](https://doi.org/10.3390/app9091723)) | China | Short-Term Load Fo Deep Learning LSTM Recurrent Neural Network Model for Prediction of Electric Vehicle Charging Demand. recasting for Electric Vehicle Charging Stations Based on Deep Learning Approaches. |
| David Chunhu Li, Javio Renja Felix, Yi-Ling Chin, Leonard Valentino Jusuf, Louis Jason Susanto ([2024](https://doi.org/10.3390/app14114354)) | Taiwan | Integrated Extended Kalman Filter and Deep Learning Platform for Electric Vehicle Battery Health Prediction. |
| Ding Liu,Peng Zeng,Shijie Cui, Chunhe Song ([2023](https://doi.org/10.3390/s23031618)) | China | Deep Reinforcement Learning for Charging Scheduling of Electric Vehicles Considering Distribution Network Voltage Stability. |
| Halid Kaplan, Kambiz Tehrani, Mo Jamshidi ([2021](https://doi.org/10.3390/en14206599)) | USA | A Fault Diagnosis Design Based on Deep Learning Approach for Electric Vehicle Applications. |
| Huifang Kong, Jiapeng Yan, Hai Wang, Lei Fan ([2020](https://doi.org/10.1007/s00521-019-04556-4)) | China | Energy management strategy for electric vehicles based on deep *Q*-learning using Bayesian optimization. |
| Abdul Latif, Ibrahim M. Mehedi, Mahendiran T. Vellingiri, Rahtul Jannat Meem, Thangam Palaniswamy ([2023](https://doi.org/10.3390/axioms12090852)) | Saudi Arabia | Enhanced Remora Optimization with Deep Learning Model for Intelligent PMSM Drives Temperature Prediction in Electric Vehicles. |
| Yongxiang Xia, Zhongyi Cheng, Jiaqi Zhang, Xi Chen ([2025](https://doi.org/10.3390/wevj16030184)) | China | User Cost Minimization and Load Balancing for Multiple Electric Vehicle Charging Stations Based on Deep Reinforcement Learning. |
| Ali Can Erüst, Fatma Yıldız Taşcıkaraoğlu ([2024](https://doi.org/10.1049/itr2.12588)) | Turkey | Spatio‐temporal dynamic navigation for electric vehicle charging using deep reinforcement learning. |
| Yanjun Li, Guodong Yin, Weichao Zhuang, Ning Zhang, Jinxiang Wang, Keke Geng ([2020](https://ieeexplore.ieee.org/document/8411139)) | USA | Compensating delays and noises in motion control of autonomous electric vehicles by using deep learning and unscented Kalman predictor. |
| Zhenhao Huang, Jing Wang, Xuezhong Fan, Renfeng Yue, Cheng Xiang, Shuhua Gao ([2024](https://ieeexplore.ieee.org/document/10637244)) | China | Economical electric vehicle charging scheduling via deep imitation learning. |
| Decui Liang, Xinyi Chen, Yinrunjie Zhang ([2024](https://ieeexplore.ieee.org/document/10582448)) | China | Diagnosing Electric Vehicle Failures with Long-Tailed Multilabel Consumer Reviews: A Bilateral-Branch Deep Learning Approach. |
| Lydia Douaidi, Sidi-Mohammed Senouci, Ines El Korbi, Fouzi Harrou ([2023](https://ieeexplore.ieee.org/document/10199832/)) | France | Predicting Electric vehicle charging stations occupancy: a federated deep learning framework. |
| Aby N Raj, K. Sakthivel ([2024](https://ieeexplore.ieee.org/document/10653515/)) | India | Driving into the Future: Exploring Machine Learning Approaches for Optimal Energy Management in Electric Vehicles: A Review of Challenges and Future Recommendations. |
| Niu Zegong, He Hongwen, Wang Yong, Huang Ruchen ([2022](https://ieeexplore.ieee.org/document/9835049/)) | China | Energy Management Optimization for Connected Hybrid Electric Vehicle with Offline Reinforcement Learning. |
| Yuge Duan, Zhao Liu, Xihao Wang, Xiaojun Wang, Qingkai Sun, Zhiyi Zhao ([2023](https://ieeexplore.ieee.org/document/10166611/)) | China | Deep Reinforcement Learning-based Electric Vehicle Charging Optimization with Renewable Generations. |
| Sanjeev Kumar Shah, Mahipal Singh ([2023](https://ieeexplore.ieee.org/document/10100037/https://ieeexplore.ieee.org/document/10100037/https://ieeexplore.ieee.org/document/10100037/https://ieeexplore.ieee.org/document/10100037/https://ieeexplore.ieee.org/document/10100037/https://ieeexplore.ieee.org/document/10100037/https://ieeexplore.ieee.org/document/10100037/https://ieeexplore.ieee.org/document/10100037/https://ieeexplore.ieee.org/document/10100037/https://ieeexplore.ieee.org/document/10100037/https://ieeexplore.ieee.org/document/10100037/)) | India | The smart energy and power estimation of electric vehicle battery using Deep learning model. |
| Mehrshad Shokati, Parisa Mohammadi, Atoosa Amirinian ([2024](https://ieeexplore.ieee.org/document/10963615/)) | Iran | Advancements in Electric Vehicle Charging Optimization: A Survey of Reinforcement Learning Approaches. |
| Bo Lin, Bissan Ghaddar, Jatin Nathwani ([2022](https://ieeexplore.ieee.org/document/9520134)) | Canada | Deep reinforcement learning for the electric vehicle routing problem with time Windows. |
| Youssef Nait Malek, Mehdi Najib, Mohamed Bakhouya, Mohammed Essaaidi ([2021](https://ieeexplore.ieee.org/document/9321203)) | Morocco | Multivariate deep learning approach for electric vehicle speed forecasting. |
| Debottam Mukherjee, Samrat Chakraborty ([2022](https://ieeexplore.ieee.org/document/9777004/)) | India | A deep learning approach for an effective speed and torque forecasting policy of PMS motors in electric vehicles. |
| Meng Zhao, Donghe Li, Qingyu Yang ([2024](https://ieeexplore.ieee.org/document/10691857/)) | China | Electric Vehicle Charging/Discharging Control Method based on Deep Reinforcement Learning for Photovoltaic Energy Storage Stations. |
| K Madumathi, T.S. Saravanan, K. Lakshmi Khandan, R. Raja, Sathish Kannan, Rajendiran M ([2024](https://ieeexplore.ieee.org/document/10544866/)) | India | Deep learning approach for predicting output current ripple in electric vehicle chargers. |
| Sunan Hu, Xiaodong Wu, Jie Li ([2021](https://ieeexplore.ieee.org/document/9661213/)) | China | Adaptive Energy Management Strategy based on deep reinforcement learning for extended-range electric vehicles. |
| Kalaimathi B, Jayanthy S, Shivasakthi R, Sowntharyaa V, Trishna AK ([2025](https://ieeexplore.ieee.org/document/10933453/)) | India | Deep Learning-Based Battery Health monitoring and speed Control alerts in electric vehicles. |
| Arash Khalatbarisoltani, Jie Han, Wenxue Liu, Cong-zhi Liu, Xiaosong Hu ([2024](https://ieeexplore.ieee.org/document/10636783/)) | China | Health-Consciousness integrated thermal and energy management of connected hybrid electric vehicles using cooperative Multi-Agent deep reinforcement learning. |
| K. Mohana Sundaram, Azham Hussain, P. Sanjeevikumar, Jens Bo Holm-Nielsen, Vishnu Kumar Kaliappan, B. Kavya Santhoshi  ([2021](https://ieeexplore.ieee.org/document/9371691/)) | India | Deep Learning for fault diagnostics in bearings, insulators, PV panels, power lines, and Electric Vehicle Applications—The State-of-the-Art Approaches. |
| Xavier Weiss, Qianwen Xu, Lars Nordström ([2022](https://ieeexplore.ieee.org/document/9907203/)) | Sweden | Energy Management of Smart Homes with Electric Vehicles Using Deep Reinforcement Learning. |
| Jamila Hemdani, Laid Degaa, Moez Soltani, Nassim Rizoug, Achraf Jabeur Telmoudi, Abdelkader Chaari ([2022](https://ieeexplore.ieee.org/document/9804048/)) | France | Prediction of aging electric vehicle battery by multi-physics modeling and deep learning method. |
| Sangyoon Lee, Dae-Hyun Choi ([2024](https://ieeexplore.ieee.org/document/10458433/)) | South Korea | Multilevel deep reinforcement learning for secure Reservation-Based electric vehicle charging via differential privacy and energy storage system. |
| Reza Shahbazian, Francesca Guerriero ([2024](https://ieeexplore.ieee.org/document/10919844/)) | Italy | Deep reinforcement learning for Large-Scale efficient routing of electric vehicles. |
| R. Arul Jose, P. Ebby Darney, R. Santhana Krishnan, J. Relin Francis Raj, D. Jansi Rani, P. Sundaravadivel ([2025](https://ieeexplore.ieee.org/document/10968839/)) | India | A hybrid deep learning model for optimizing electric vehicle battery and navigation systems. |
| Mouaad Boulakhbar, Markos Farag, Kawtar Benabdelaziz, Malika Zazi, Mohamed Maaroufi, Tarik Kousksou ([2022](https://ieeexplore.ieee.org/document/9738115)) | France | Electric vehicles arrival and departure time prediction based on deep learning: the case of Morocco. |
| Nectar Jinil, Sofana Reka ([2019](https://ieeexplore.ieee.org/document/8719243/)) | India | Deep Learning method to predict Electric Vehicle power requirements and optimizing power distribution. |
| Arash Khalatbarisoltani, Jie Han ([2024](https://ieeexplore.ieee.org/document/10567648/)) | China | Connected Hybrid Electric Vehicles with Policy-Sharing: A Combination of Deep Reinforcement Learning and Federated Learning. |
| Zheng Mei, Jinhong Sun, Hucheng Yi ([2023](https://ieeexplore.ieee.org/document/10263056/)) | China | Modeling and performance analysis of electric vehicle air conditioning systems based on deep learning. |
| Mohammad Javad Peyravi, Mohsen Kalantar, Aref Eskandari, Sherko Salehpour ([2025](https://ieeexplore.ieee.org/document/10966125/)) | Iran | Real-Time pricing for electric vehicle charging stations using deep reinforcement learning under congestion and peak shifting. |
| Manoj Basnet, Mohd. Hasan Ali ([2020](https://ieeexplore.ieee.org/document/9243152/)) | USA | Deep learning-based intrusion detection system for electric vehicle charging station. |
| Sulaiman S. M., Judeson Antony Kovilpillai J, Mahmood Hussain Mir, Golda Brunet R, Pragya, Soumi ([2024](https://doi.org/10.1109/ICITEICS61368.2024.10624958)) | India | Battery Range Estimation in Electric Vehicles Using Machine Learning and Deep Learning Techniques. |
| Huifan Deng, Youqun Zhao, Anh-Tu Nguyen, Chao Huang ([2023](https://doi.org/10.1109/TMECH.2022.3233705)) | France | Fault-Tolerant Predictive Control with Deep-Reinforcement-Learning-Based Torque Distribution for Four In-Wheel Motor Drive Electric Vehicles. |
| Yingjiu Pan, Wenpeng Fang, Wenshan Zhang ([2023](https://doi.org/10.1016/j.jclepro.2023.136742)) | China | Development of an energy consumption prediction model for battery electric vehicles in real-world driving: A combined approach of short-trip segment division and deep learning. |
| Mekkaoui Djamel Eddine, Yanming Shen ([2022](https://doi.org/10.1007/s11227-022-04428-0)) | China | A deep learning based approach for predicting the demand of electric vehicle charge. |
| Chenyuan Liu, Heng Li, Kexin Li, Yue Wu, Baogang Lv ([2025](https://doi.org/10.3390/en18061463)) | China | Deep Learning for State of Health Estimation of Lithium-Ion Batteries in Electric Vehicles: A Systematic Review. |
| Abdul Latif, Ibrahim M. Mehedi, Mahendiran T. Vellingiri, Rahtul Jannat Meem, Thangam Palaniswamy ([2023](https://doi.org/10.3390/axioms12090852)) | Saudi Arabia | Enhanced Remora Optimization with Deep Learning Model for Intelligent PMSM Drives Temperature Prediction in Electric Vehicles. |
| Heejun Kwon, Sugi Choi, Wonmyung Woo, Haiyoung Jung ([2025](https://doi.org/10.3390/fire8020066)) | South Korea | Evaluating Segmentation-Based Deep Learning Models for Real-Time Electric Vehicle Fire Detection. |
| T. Blesslin Sheeba, C. Sharanya, C. Nayanatara, S. K. Indumathi, K. Kalins, G. Ignisha Rajathi  ([2022](https://doi.org/10.1063/5.0109161)) | India | Deep learning enabled smart charging technology for electric vehicles. |
| Marina Dorokhova, Yann Martinson, [Christophe Ballif](https://www.sciencedirect.com/author/6701805412/christophe-ballif), Nicolas Wyrsch ([2021](https://doi.org/10.1016/j.apenergy.2021.117504)) | Switzerland | Deep reinforcement learning control of electric vehicle charging in the presence of photovoltaic generation. |
| Changcheng Wu, Jiankun Peng, Hongwen He,  Jiageng Ruan, Jun Chen, Chunye Ma ([2024](https://doi.org/10.1016/j.jpowsour.2024.235463')) | China | Health-awareness energy management strategy for battery electric vehicles based on self-attention deep reinforcement learning. |
| De Zhao, Hua Wang, Zhiyuan Liu ([2022](https://doi.org/10.1155/2022/4372168)) | China | Charging-Related state prediction for electric vehicles using the deep learning model. |
| Vijay Kumar, Vaibhav Jain ([2024](https://doi.org/10.1002/est2.70072)) | India | A deep learning dependent controller for advanced ultracapacitor SOC concept to increase battery life span of electric vehicles. |
| Hongqian Wei, Wenqiang Zhao, Qiang Ai, Youtong Zhang, Tianyi Huang ([2022](https://doi.org/10.1049/itr2.12176)) | China | Deep reinforcement learning based active safety control for distributed drive electric vehicles. |
| Ali Jamali Jahromi, Mohammad Reza Masoudi, Mohammad Mohammadi, Shahabodin Afrasiabi ([2024](https://doi.org/10.1049/gtd2.13276)) | Iran | Improving electric vehicle charging forecasting: A hybrid deep learning approach for probabilistic predictions. |
| Weihan Li, Han Cui, Thomas Nemeth, Jonathan Jansen, Cem Ünlübayir, Zhongbao Wei, Lei Zhang, Zhenpo Wang, Jiageng Ruan, Haifeng Dai, Xuezhe Wei, [Dirk Uwe Sauer](https://www.sciencedirect.com/author/7005040792/dirk-uwe-sauer)  ([2021](https://doi.org/10.1016/j.est.2021.102355)) | Germany | Deep reinforcement learning-based energy management of hybrid battery systems in electric vehicles. |
| Shahid A. Hasib, Muhammad Majid Gulzar, Adnan Shakoor, Salman Habib, Ali Faisal Murtaza ([2024](https://doi.org/10.1016/j.rineng.2024.103630)) | France | Optimizing electric vehicle driving range prediction using deep learning: A deep neural network (DNN) approach. |
| Xuefeng Han, Hongwen He, Jingda Wu, Jiankun Peng, Yuecheng Li ([2019](https://doi.org/10.1016/j.apenergy.2019.113708)) | China | Energy management based on reinforcement learning with double deep Q-learning for a hybrid electric tracked vehicle. |
| Hongwen He, Jianfei Cao,  Xing Cui ([2020](https://doi.org/10.1016/j.jclepro.2019.119302)) | China | Energy optimization of electric vehicle’s acceleration process based on reinforcement learning. |
| Anupong Wongchai, Yassine Aoudni, Mannava Yesubabu, Faheem Ahmad Reegu, N. Vasantha Gowri, [P. Vijayakumar](https://www.sciencedirect.com/author/55975531200/vijayakumar-peroumal) ([2023](https://doi.org/10.1016/j.seta.2023.103178)) | Thailand | Sustainable electric vehicles fault detection based on monitoring by deep Learning Architectures in feature extraction and classification. |
| Dawei Zhang, Chen Zhong,Peijuan Xu, Yiyang Tian ([2022](https://doi.org/10.3390/machines10100912)) | China | Deep Learning in the State of Charge Estimation for Li-Ion Batteries of Electric Vehicles: A Review. |
| Sahar Koohfar, Wubeshet Woldemariam, Amit Kumar ([2023](https://doi.org/10.3390/su15032105)) | USA | Prediction of Electric Vehicles Charging Demand: A Transformer-Based Deep Learning Approach. |
| Venkata Satya Rahul Kosuru, Ashwin Kavasseri Venkitaraman ([2023](https://doi.org/10.3390/wevj14040101)) | USA | A Smart Battery Management System for Electric Vehicles Using Deep Learning-Based Sensor Fault Detection. |
| Mihir Trivedi, Riya Kakkar, Rajesh Gupta,Smita Agrawal, Sudeep Tanwar, Violeta-Carolina Niculescu, Maria Simona Raboaca, Fayez Alqahtani, Aldosary Saad, Amr Tolba ([2022](https://doi.org/10.3390/math10193626)) | India | Blockchain and Deep Learning-Based Fault Detection Framework for Electric Vehicles. |
| Gayathry Vishnu, Deepa Kaliyaperumal, Peeta Basa Pati, Alagar Karthick, Nagesh Subbanna, Aritra Ghosh ([2023](https://doi.org/10.3390/wevj14090266)) | India | Short-Term Forecasting of Electric Vehicle Load Using Time Series, Machine Learning, and Deep Learning Techniques. |
| David Chunhu Li, Javio Renja Felix,Yi-Ling Chin, Leonard Valentino Jusuf, Louis Jason Susanto ([2024](https://doi.org/10.3390/app14114354)) | Taiwan | Integrated Extended Kalman Filter and Deep Learning Platform for Electric Vehicle Battery Health Prediction. |
| Prince Aduama, Zhibo Zhang, Ameena S. Al-Sumaiti ([2023](https://doi.org/10.3390/en16031309)) | UAE | Multi-Feature Data Fusion-Based Load Forecasting of Electric Vehicle Charging Stations Using a Deep Learning Model. |
| Sooji Ha, Daniel J. Marchetto,  Sameer Dharur, Omar I. Asensio ([2021](https://doi.org/10.1016/j.patter.2020.100195)) | USA | Topic Classification of electric vehicle consumer Experiences with transformer-based deep learning. |
| Raid Mohsen Alhazmi ([2024](https://doi.org/10.3390/wevj15090385)) | Saudi Arabia | State of Health Prediction in Electric Vehicle Batteries Using a Deep Learning Model. |
| Bishoy E. Sedhom, Abdelfattah A. Eladl, Pierluigi Siano, Magda I. El-Afifi ([2025](https://www.sciencedirect.com/science/article/pii/S0142061525000961)) | Egypt | Dual-layered deep learning and optimization algorithm for electric vehicles charging infrastructure planning |
| Tallataf Rasheed, Abdul Rauf Bhatti, Muhammad Farhan, Akhtar Rasool, Tarek H. M. El-Fouly ([2023](https://ieeexplore.ieee.org/abstract/document/10225522)) | Pakistan | Improving the Efficiency of Deep Learning Models Using Supervised Approach for Load Forecasting of Electric Vehicles |
| Aayushi Priyadarshini, Shekhar Yadav, Nitesh Tiwari ([2025](https://www.sciencedirect.com/science/article/pii/S2772671125000828)) | India | Smart multioutput fast charger for electric vehicles using deep learning controllers |
| Fei Ren, Chenlu Tian, Guiqing Zhang, Chengdong Li, Yuan Zhai ([2022](https://www.sciencedirect.com/science/article/pii/S0360544222006417?casa_token=8apvcWthng0AAAAA:y_rAvatUTAZUSMrv4cYv5G0-l2mc1Ovk7kOsdOo9VLruQLD6VvHPuUXtFJoaTr7HBikI_31oXBA)) | China | A hybrid method for power demand prediction of electric vehicles based on SARIMA and deep learning with integration of periodic features |
| Yanjun Li, Guodong Yin, Weichao Zhuang, Ning Zhang, Jinxiang Wang, Keke Geng ([2018](https://ieeexplore.ieee.org/abstract/document/8411139?casa_token=NClc6f8qhaoAAAAA:j75Qadnpi4fY52y4UH9kkdYx1OJNFSNUX5WqxmbiuPWniHP11FCJPf5ifb8ePlViD0lqMGjjuw)) | USA | Compensating Delays and Noises in Motion Control of Autonomous Electric Vehicles by Using Deep Learning and Unscented Kalman Predictor |
| Halid Kaplan, Kambiz Tehrani, Mo Jamshidi ([2021](https://www.mdpi.com/1996-1073/14/20/6599)) | USA | A Fault Diagnosis Design Based on Deep Learning Approach for Electric Vehicle Applications |
| Dona George, Sivraj P ([2021](https://ieeexplore.ieee.org/abstract/document/9532912?casa_token=nhkWd1GkVhIAAAAA:HYDcHH0kLeDLlvLspab0AI64TTT1B8HzG22FzA6Nu9hEeUlqD-JnyeKtj9mH14WG8lvRARS0Hg)) | India | Driving Range Estimation of Electric Vehicles using Deep Learning |
| Jinpeng Tian Jahangir, Rui Xiong, Weixiang Shen, Jiahuan Lu ([2021](https://www.sciencedirect.com/science/article/pii/S0306261921003147)) | China | State-of-charge estimation of LiFePO4 batteries in electric vehicles: A deep-learning enabled approach |
| Hamidreza Jahangir, Saleh Sadeghi Gougheri, Behzad Vatandoust, Masoud Aliakbar Golkar, Ali Ahmadian, Amin Hajizadeh ([2020](https://ieeexplore.ieee.org/abstract/document/9102300?casa_token=TYT8pzRjduwAAAAA:30JJQ98ZlkXsfK6rE04M918nebW1Va0vO2d5vE0oKNHJPBwuB5XI5y3C_XYXl2oZGpC3FyMIfQ)) | Iran | Plug-in Electric Vehicle Behavior Modeling in Energy Market: A Novel Deep Learning-Based Approach With Clustering Technique |
| Xilei Sun, Jianqin Fu, Huiyong Yang, Mingke Xie, Jingping Liu ([2023](https://www.sciencedirect.com/science/article/pii/S0360544223001664?casa_token=LLMG4AELx-0AAAAA:L3npyZKVmqhvXPcQH3JEppt_Db879tPo6n8EpVSYCvIdZyp_Kl0VIySfL92_9J3kDlu_HFpavWA)) | China | An energy management strategy for plug-in hybrid electric vehicles based on deep learning and improved model predictive control |
| Seyedeh Mahsa Sotoudeh, Baisravan HomChaudhuri ([2023](https://ieeexplore.ieee.org/abstract/document/10004938/?casa_token=N5-eHLoVUgEAAAAA:5tYQKmLV0ZYpu7X0CfDIvIqzLs1CwiO82CkbinFhxrOGm-DOpfRzYsyOG2dflAIByFuH1FrRKQ)) | USA | A Deep-Learning-Based Approach to Eco-Driving-Based Energy Management of Hybrid Electric Vehicles |
| Shengyou Wang, Chengxiang Zhuge, Chunfu Shao, Pinxi Wang, Xiong Yang, Shiqi Wang ([2023](https://www.sciencedirect.com/science/article/pii/S0306261923003963?casa_token=TQH0W7tE8z4AAAAA:3SEZ7oRhBEO7exXEcyBiw-uGOUtyZqUyl01HUv6OixNEPgjSU5CQVm2SUt3CL5AM4cBA1QqxSx8)) | China | Short-term electric vehicle charging demand prediction: A deep learning approach |
| H. Pourvaziri, H. Sarhadi, N. Azad, H. Afshari, M. Taghavi ([2024](https://www.sciencedirect.com/science/article/pii/S1366554524001595)) | Canada | Planning of electric vehicle charging stations: An integrated deep learning and queueing theory approach |
| Fallah Saad El, Kharbach Jaouad, Vanagas Jonas, Vilkelyte Zivile, Tolavaisiene Sonata, Ikmel Ghita, Redouani Assia, Lagnaoui Saloua, Boumais Khaoula, Jamil Mohammed Ouazzani([2024](https://www.engineeringvillage.com/app/doc/?docid=cpx_3147400419049a69199M677f10178165134&pageSize=25&index=110&searchId=fe3d73bdec7a40a3a274ed88f853d0b2&resultsCount=19398&usageZone=resultslist&usageOrigin=searchresults&searchType=Expert)) | Morocco | Advanced Battery Management for Electric Vehicles: A Deep Dive into Estimation Techniques Based on Deep Learning for the State of Health and State of Charge of Lithium-Ion Batteries |
| Seyed Amir Hosseini, Behrooz Taheri, Seyed Hossein Hesamedin Sadeghi, Adel Nasiri ([2024](https://www.sciencedirect.com/science/article/pii/S2772671124004248)) | Iran | A deep learning model for fault detection in distribution networks with high penetration of electric vehicle chargers |
